# Informing Optimal Environmental Influenza Interventions: How the Host, Agent, and Environment Alter Dominant Routes of Transmission

**DOI:** 10.1371/journal.pcbi.1000969

**Published:** 2010-10-28

**Authors:** Ian H. Spicknall, James S. Koopman, Mark Nicas, Josep M. Pujol, Sheng Li, Joseph N. S. Eisenberg

**Affiliations:** 1 Department of Epidemiology, University of Michigan, Ann Arbor, Michigan, United States of America; 2 Department of Environmental Health Sciences, University of California, Berkeley, Berkeley, California, United States of America; 3 Telefonica, Catalonia, Spain; Imperial College London, United Kingdom

## Abstract

Influenza can be transmitted through respirable (small airborne particles), inspirable (intermediate size), direct-droplet-spray, and contact modes. How these modes are affected by features of the virus strain (infectivity, survivability, transferability, or shedding profiles), host population (behavior, susceptibility, or shedding profiles), and environment (host density, surface area to volume ratios, or host movement patterns) have only recently come under investigation. A discrete-event, continuous-time, stochastic transmission model was constructed to analyze the environmental processes through which a virus passes from one person to another via different transmission modes, and explore which factors increase or decrease different modes of transmission. With the exception of the inspiratory route, each route on its own can cause high transmission in isolation of other modes. Mode-specific transmission was highly sensitive to parameter values. For example, droplet and respirable transmission usually required high host density, while the contact route had no such requirement. Depending on the specific context, one or more modes may be sufficient to cause high transmission, while in other contexts no transmission may result. Because of this, when making intervention decisions that involve blocking environmental pathways, generic recommendations applied indiscriminately may be ineffective; instead intervention choice should be contextualized, depending on the specific features of people, virus strain, or venue in question.

## Author Summary

We model the transmission of influenza through the environment assuming four possible transmission routes: respiratory (small particle inhalation), inspiratory (medium particle inhalation), direct-droplet-spray (large particle spray directly to susceptible tissue), and contact-mediated (when large particles settle in the environment, are picked up, and self-inoculated). There is much disagreement in the literature with regard to the dominant route of influenza transmission. Using empirical estimates where possible, we vary 18 parameters which are relevant to these transmission routes. These parameters are features of the agent, host, or environment. Depending on these features, a specific route or routes may be operating at a high intensity. Thus, it is unlikely there is a single universal dominant influenza transmission route. Therefore, interventions which target only one of these routes will not be optimal in all settings. It is important to understand the context in terms of the agent, host, and environment in order to develop optimal environmental intervention strategies.

## Introduction

On June 11, 2009 the WHO declared the H1N1 influenza virus a pandemic. Health organizations worldwide were prompted to escalate their efforts to minimize transmission within their jurisdictions. Airports began to monitor incoming passengers while schools increased their already intensive surveillance activities. Recommendations were established with regard to masks, hygiene, decontamination, and isolation of suspected cases. This interest in intervention and control of person-to-person transmitted illnesses with multiple potential routes of transmission began to intensify during the emergence of SARS and later the H5N1 (avian influenza) virus. Heightened awareness of the potential for another pandemic influenza led to increased funding to study non-pharmaceutical interventions by the CDC as well as increased efforts in modeling influenza transmission. These studies were funded in order to better understand optimal intervention and control strategies. Much insight was gained into influenza mitigation strategies such as border closure, social distancing, antiviral prophylaxis, restriction of public transportation, and school closure [1]–[8]. To date, however, little is known about the relative contributions of the different influenza transmission modes and how these might vary due to heterogeneity in viral strain, host, and environment.

This manuscript explores potential effects of these unknown factors by presenting: 1) a transmission model structure that explicitly describes the environmental processes through which viruses pass from one person to another, thereby distinguishing the different modes of transmission; and 2) an analytical approach that explores which factors increase or decrease different modes of transmission under the given model structure. The model analyzed is an environmental infection transmission system model that elaborates the approach to such models by Li et al. [9] by formulating the model in a discrete event framework and greatly expanding on the details of the various processes involved. It does not define contact events with transmission probabilities for each event as most transmission models do [10]. A problem with that approach is defining what constitutes a contact. Instead we define events related to virus excretion, environmental survival, uptake, and causation of infection. This allows us to address events at a level that is more relevant to possible interventions and the construction of more meaningful causal theory.

To inform relevant intervention options for influenza, we consider four potential modes of transmission: respirable, inspirable, direct-droplet-spray, and contact mediated transmission [11]–[13]. In this manuscript we consider each mode as follows. Respirable transmission occurs when viruses on small particles (<10 µm diameter) are inhaled and deposit in the alveolar region of the lower respiratory tract. Inspirable transmission occurs when viruses on medium size particles (>10 and <100 µm diameter) are inhaled and deposit in the upper respiratory tract. Direct-droplet-spray transmission (hereafter referred to as droplet transmission) occurs when viruses on large particles (>100 µm diameter) from the cough or sneeze of an infected individual deposit directly on a susceptible individual's mucous membranes. Contact transmission occurs when an infected person contaminates their own hands or contaminates surfaces via their hands or via droplets with virus laden large particles. Transfer of pathogens may then result in contamination of the hands of others who then may touch their eyes, nose or mouth to self-inoculate, potentially infecting the upper respiratory tract. We assess how different feasible model parameters influence how much transmission follows these different routes.

**Figure 1:**
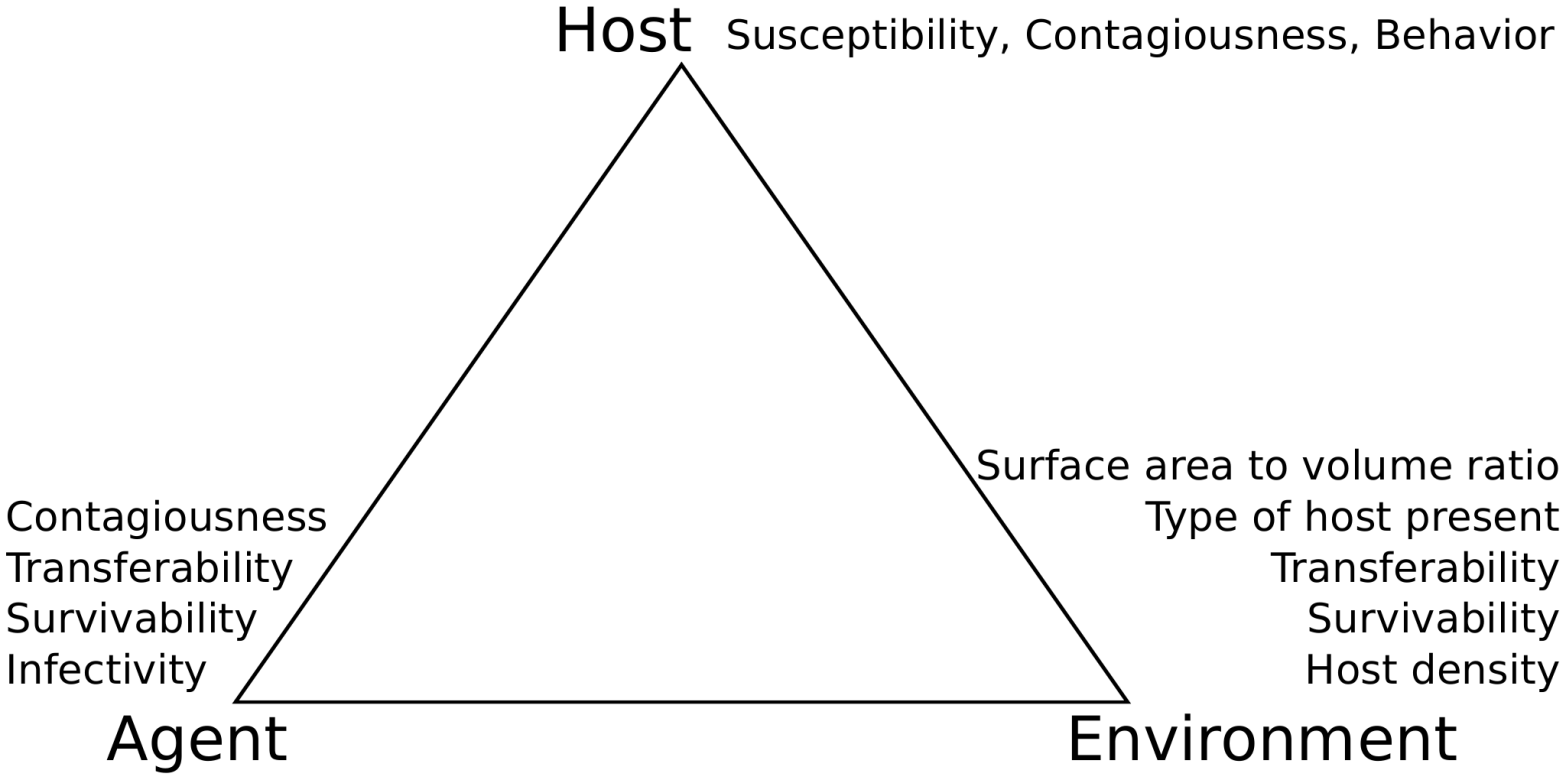
The epidemiologic triad for environmentally mediated influenza transmission. Specific features are listed in each corner that are relevant to either the agent (specific virus strain), host, and environmental venue.

For example, different viruses may have different infectivity, survivability, transferability, or shedding profiles. Similarly, among different populations who have different behaviors, susceptibility profiles, or shedding profiles, the same virus may have different effects depending on the type of population present. Finally, even with identical viral strains and human populations, environmental venues may have variable host densities, surface area to volume ratios, or host movement patterns that can generate different population level infection outcomes. These diverse sources of heterogeneity that we address form the corners of the epidemiologic triad (figure 1).

We assess the effects of these sources of heterogeneity on relative magnitude of influenza transmission modes in a scenario where all individuals move randomly in an identical fashion. We construct a detailed stochastic individual based model of environmental influenza transmission. We use values from empirical literature as well as expert judgment to parameterize this model. We apply upper and lower parameter constraints to 18 parameters, and obtain a Latin hypercube sample of this constrained parameter space. We analyze the resulting outcome space with respect to how different transmission modes are more or less important in specific contexts.

With this work we contribute to the body of literature discussing the dominant mode of influenza transmission [12], [14]–[18]. Additionally, this work takes an incremental step forward from previous environmental infection transmission models [7], [9], [19]–[21] as: 1) we model all four modes of influenza transmission simultaneously; 2) we do so in an agent based framework rather than with ordinary differential equation based framework; and 3) this model is solely informed parametrically by empirical work—no model fitting or optimization procedures were used to parameterize this model. We explicitly point out where the holes in the empirical literature exist. We show that depending on the scenario, one mode may be more or less important than another. Therefore, when intervening, generic recommendations applied indiscriminately may be ineffective; instead intervention choice should be contextualized depending on the specific features of people, virus, or venue in question. We consider how features related to pathology, behavior, and microbiology in the host, pathogen, and environment (figure 1) alter the magnitude of transmission via each mode.

## Materials and Methods

### The model

We model environmental influenza transmission in a venue by considering infections resulting from contact-mediated, respirable, inspirable, and droplet exposures. We model a single uniform abstract venue with no variation in space with regard to fomites or behavior in order to seek simple general insights. This venue homogeneity helps us identify sources of heterogeneity in transmission attributable to the factors we study in the epidemiological triad (figure 1). The venue is described as a lattice grid with discrete cell locations which people visit. Each cell in the lattice has a surface area, given by its length and width (2 meters by 2 meters), and local air volume, resulting in a surface area to volume ratio.

**Figure 2:**
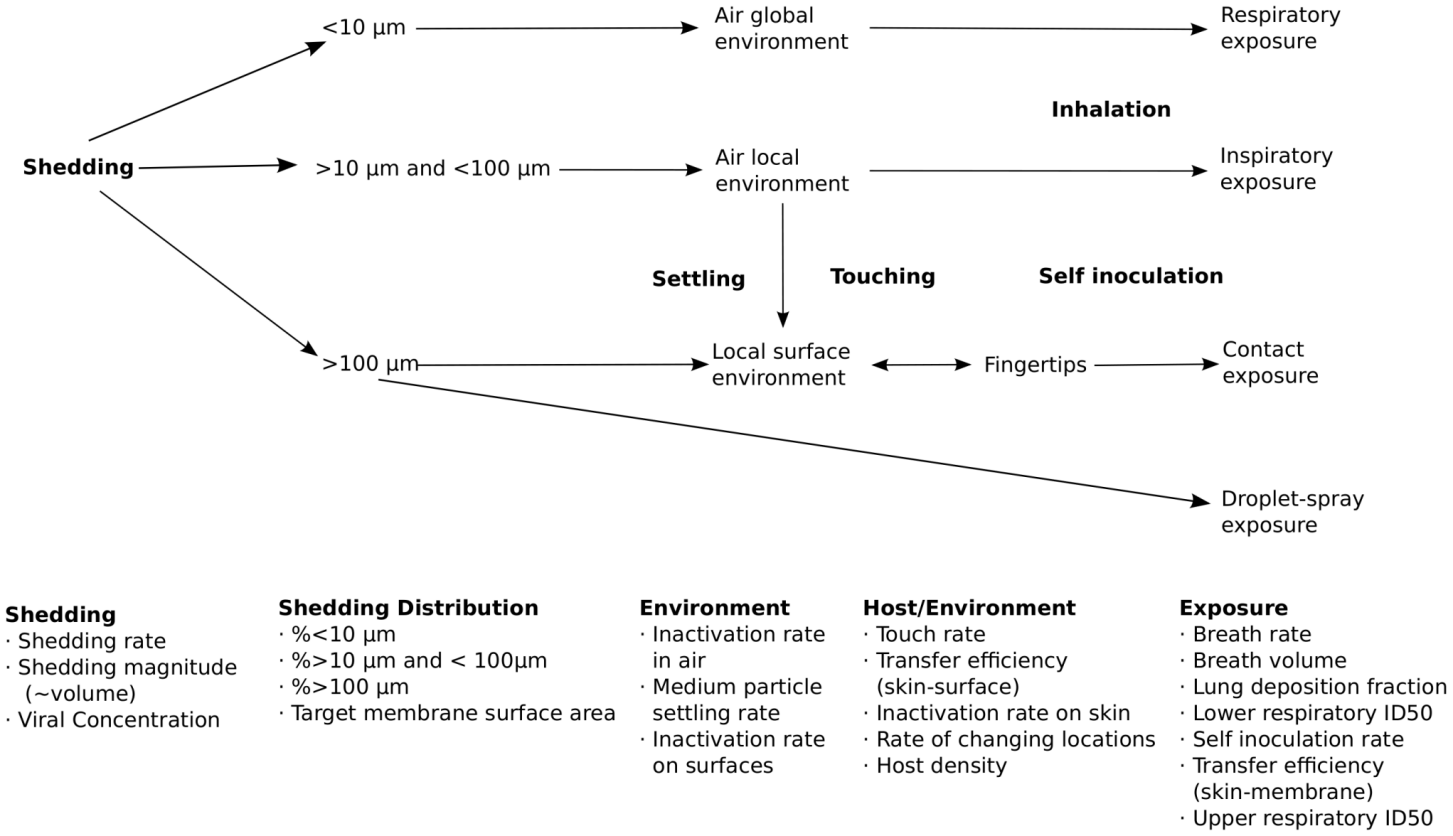
Schematic of pathogen flow through the environment with specific events in bold resulting in respiratory, inspiratory, contact or droplet exposure. Relevant governing parameters of transmission are listed below each phase. Viral inactivation occurs in the air, on surfaces, and on fingertips (not explicitly shown). Moving from the left to the right of the diagram, viral excretion magnitude is determined by the shedding rate, volume, and concentration. Where these viruses go is determined by the size of the particle they adhere to during excretion. Based on cough particle size distribution data, these are divided proportionally. Viruses on small particles are well mixed, and are assumed to either inactivate or be inhaled (respiratory exposure) before settling would occur. Viruses on medium particles may either inactivate, settle to the local surfaces, or be inhaled (inspiratory exposure). Some viruses on large particles may be utilized initially in droplet exposure, proportional to the target facial membrane surface area multiplied by the number of susceptible collocated with the shedder. Viruses on larger particles not utilized in droplet exposure is assumed to settle immediately to the local surface environment. Here it may inactivate, or be picked up on fingertips. Once on fingertips, the virus may inactivate, be deposited back to a surface environment, or be used in contact exposure via self-inoculation. Respiratory exposure assumes lower respiratory penetration and uses an ID50 specific to this region. Inspiratory, droplet, and contact exposure assumes the potential for infection only occurs in the upper respiratory tract and all use the same ID50 specific to this region. For simplicity, we assume exponential dose-response relationship.

Figure 2 provides a schematic of all processes resulting from each shedding event that lead to exposure. We use continuous time to model discrete spatial units, humans, pathogens, and transmission-related events. Transmission-related events are described in the caption of figure 2 and in greater detail in the online material. An infectious individual sheds virus as a function of a shedding rate (a cough rate), shedding magnitude (how much mucous volume is put out), and viral concentration of material being excreted. Together, this determines the number of virus particles excreted. Next, particles are categorized by the relative weights of cough particles: <10µm; between >10µm and <100µm; and >100µm. Note that we assume the same viral concentration regardless of particle size. We assume that only virus on particles >100µm may cause droplet exposure if there are individuals collocated with the shedder. We assume that all viruses on particles <10µm are instantly and thoroughly mixed throughout the venue by invoking the well mixed room assumption for these small particles. We assume these remain aerosolized until either the virus inactivates, leaves the venue due to air exchanges, or is utilized in respiratory exposure in the lung alveoli.

We assume virus on particles >10µm and <100µm remain in the local environment of the shedder because these particles would be too large to invoke the well mixed room assumption. These may inactivate, settle to the local surface environment, or result in inspiratory exposure in the upper respiratory tract. These particles are too large to penetrate to the lung alveoli.

We assume particles >100µm that are not involved in droplet exposure settle immediately to the shedder's local surface environment evenly spread. Here, the virus may inactivate, be picked up as people touch this surface, and then generate contact exposure via self-inoculation. For the sake of simplicity, we assume that no excreted virus adheres to the shedder's hands (as might happen if a cough or sneeze were covered with a hand). For greater model detail refer to the supporting material.

### Sampling and simulation

We vary 18 parameters relevant to influenza transmission related to the host, pathogen, and venue (Table 1). We define a median value, either taken from the literature or from expert judgment, and either apply symmetric constraints or constraints that are symmetric when observed after a log transform, so that half of the sampled values are below the defined median and half above. We sample from the constrained parameter space using Latin hypercube sampling with uniform probability distributions for each parameter. In our full Latin hypercube sample, there are 10,000 unique parameter sets defined by the values of the 18 varied parameters. For each parameter set, 500 independent simulation trials are conducted and averaged.

**Table 1:** Parameter sampling constraints used to generate a 10,000 unit Latin hypercube sample.

Parameter	Description	Unit	Lower Constraint^1^	Upper Constraint^1^	Resulting Median^2^	Reference
μ_A_	Inactivation rate–air	Min^−1^	0.001	0.036	0.0060	[31]
μ_S_	Inactivation rate–surfaces	Min^−1^	0.0005	0.2	0.010	[32]
μ_H_	Inactivation rate–hands	Min^−1^	0.62	1.22	0.92	[32]
τ_S-H-S_	Transfer efficiency (surface to hand to surface)		0.0167	0.6	0.10	[33], [34]
τ_F-T_	Transfer proportion (eyes/nose/mouth to target mucous membranes)		0.05	0.25	0.15	
τ_L_	Lung deposition fraction		0.083	0.75	0.42	[35]
π_L_	Lower respiratory HID_50_	TCID_50_	0.067	6.7	0.67	[36]
π_U_	Upper respiratory HID_50_	TCID_50_	50	5000	500	[37], [38]
α_Mag_	Shedding magnitude		0.005	0.075	0.019	
α_Resp_	Viral proportion to respirable air		1.4E-7	1.4E-5	1.4E-6	[39], [40]
α_Insp_	Viral proportion to inspirable air		0.00353	0.016	0.0095	[39], [40]
ρ_Inoc_	Rate of self inoculation	Min^−1^	0.02	0.32	0.080	[26], [27]
ρ_touch_	Rate of surface touching	Min^−1^	0.19	3	0.75	
ρ_move_	Rate of changing location	Min^−1^	0.00083	3	0.050	
ρ_breath_	Rate of breathing	Min^−1^	10	22	16	[41]
ε_settle_	Medium particle settling rate	Min^−1^	4.6	11	7.6	
ε_SA∶V_	Surface area to volume ratio	m^2^∶m^3^	1	5	3.0	[28]–[30]
ε_density_	Host density	people/m^2^	0.056	5.6	0.2	

NOTE: HID_50_ = quantity of virus required to cause infection in 50% of humans.

1 Either symmetric constraints or constraints which were symmetric when observed after a log transform were applied, so that half of the sampled values would be below the defined median and half above.

2 Median values were defined either from the literature or from expert judgment. We sampled from the constrained parameter space using Latin hypercube sampling with uniform probability distributions for each parameter.

For each trial, we use a special simulation design: when each new infection takes place, that individual is immediately replaced with a new susceptible in their place. This allows us to observe directly the number of new infections transmitted from one infected person over the course of their infection in the presence of a completely susceptible population of constant size—which is one definition of the basic reproductive number, R_0_ [22], [23]. Additionally, we are able to differentiate whether infection takes place from one mode or another, allowing us to directly observe mode-specific R_0_'s.

### Statistical analyses

To examine transmission mode dominance we categorize regions of the full 10,000 unit space into regions where one or more transmission modes have a mode-specific R_0_ above 1.7 (a plausible value of the 1918 influenza pandemic R_0_ [1]). We also considered using a cut-point of 1.2, but all results were similar and for simplicity not shown. We visualize this with a Venn diagram, and use box-plots to compare the parameter distributions of each category to one another. To examine parameters which affected each transmission mode intensity, we perform a simple correlation analysis (presented in the supporting material) and use the classification and regression tree (CART) algorithm [24], [25]. The CART approach classifies parameter sets as those which lead to a mode-specific R_0_ greater than 1.7, versus those less than 1.7. A tree structure is produced in which classification criteria are specified by subdivisions of parameter values.

## Results

Aggregated over all 10,000 parameter sets, the contact mode has the highest average mode-specific R_0_, 1.7. The droplet, respiratory, and inspiratory routes followed with mode-specific R_0_'s 0.27, 0.05, and 0.006 respectively. While this aggregate measure is often all that is reported in the literature, it ignores the heterogeneous effects of different contexts in inducing shifts in transmission mode dominance and intensity; that is to say, contact transmission is not necessarily dominant in all settings.

### Transmission mode dominance

**Figure 3:**
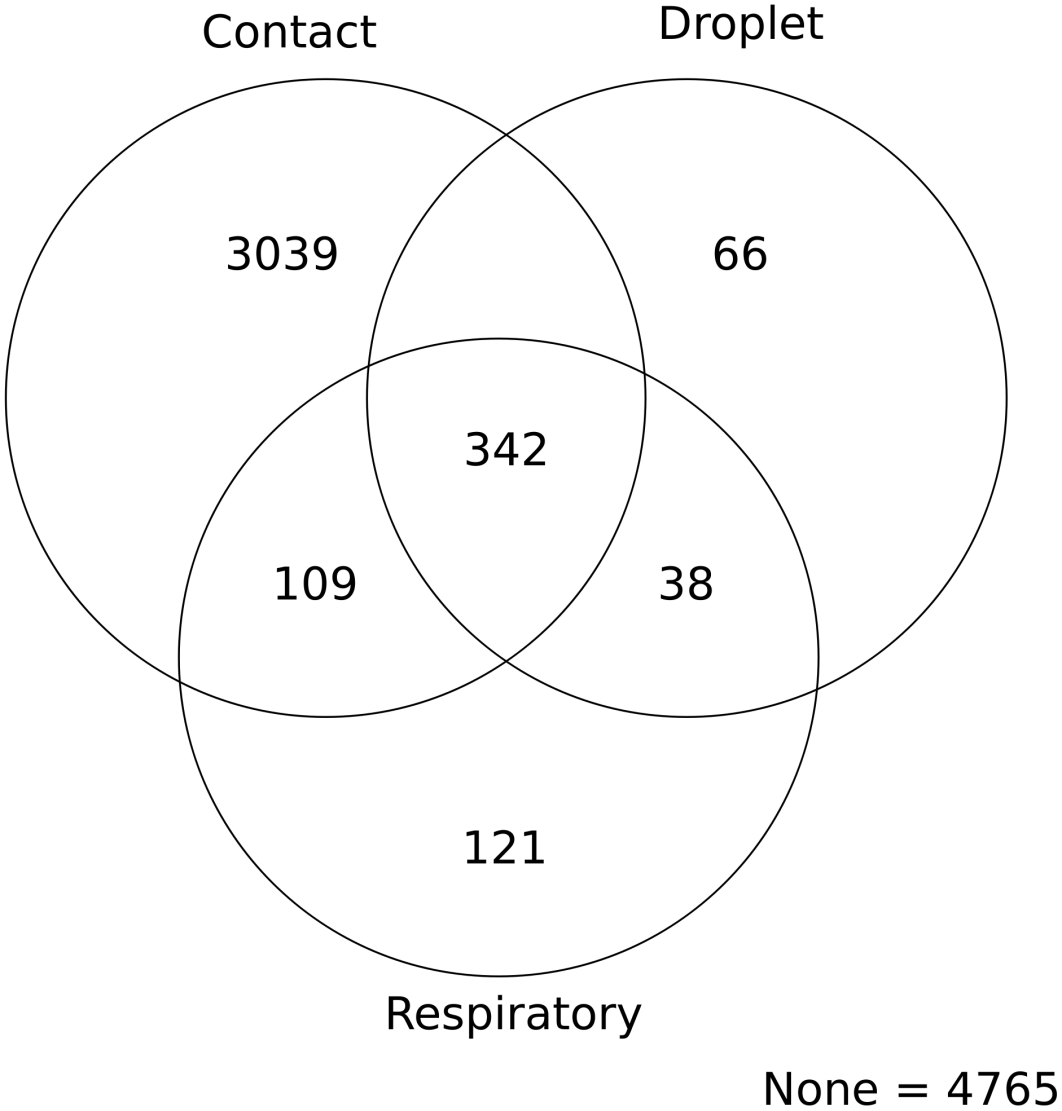
Venn diagram of influenza transmission mode dominance. Numbers in different regions reflect the number of parameter sets which yield mode-specific R_0_>1.7. Overlap indicates that more than one transmission mode has a mode-specific R_0_>1.7. The 4765 parameter sets outside these three categories indicate that none of these three modes had high mode-specific transmission in these parameter sets. Note, that of these 4765 parameter sets with no single dominant mode, 577 parameter sets still yielded a total-R_0_>1.7 when summed across all modes. The inspiratory transmission mode did not yield any parameter sets in which it alone dominated, and only 26 parameter sets in which it ever had mode-specific R_0_>1.7.

We divide the entire 10,000 unit space into mutually exclusive categories based on whether one or more transmission modes individually have a mode-specific R_0_>1.7. The contact, respiratory, and droplet transmission routes all have parameter sets which yield high transmission (mode-specific R_0_>1.7) via each mode in isolation of all other modes. There are 3079 sets where contact was high with nothing else, 121 for the respiratory mode, and 66 for droplet (figure 3). There is no parameter set in which the inspiratory mode alone was above 1.7. Each of these domains is determined by features of the host, virus, and environment, in which any of these three modes would dominate over the others.

Additionally, there was considerable overlap, where multiple modes each have a mode-specific R_0_>1.7. In these 1969 parameter sets no single mode dominates over the other modes; rather multiple modes transmit at a high intensity simultaneously. Our analysis henceforth ignores the inspiratory route as it only caused high transmission in 26 parameter sets, never occurring alone. The extent of overlap differs by transmission mode (figure 3). The droplet route has the most overlap as 96% of parameter sets that yield high droplet transmission also yield high transmission by at least one other route. 80% of parameter sets which yield high respiratory transmission also yield high transmission by at least one other mode. The contact mode is the most isolated, as only 40% of its high transmission parameter sets also yield high transmission by other modes In 4765 parameter sets, no individual mode has a mode-specific R_0_>1.7. Of these, there are 577 parameter sets which, when summed across all modes, yields a total-R0>1.7.

**Figure 4:**
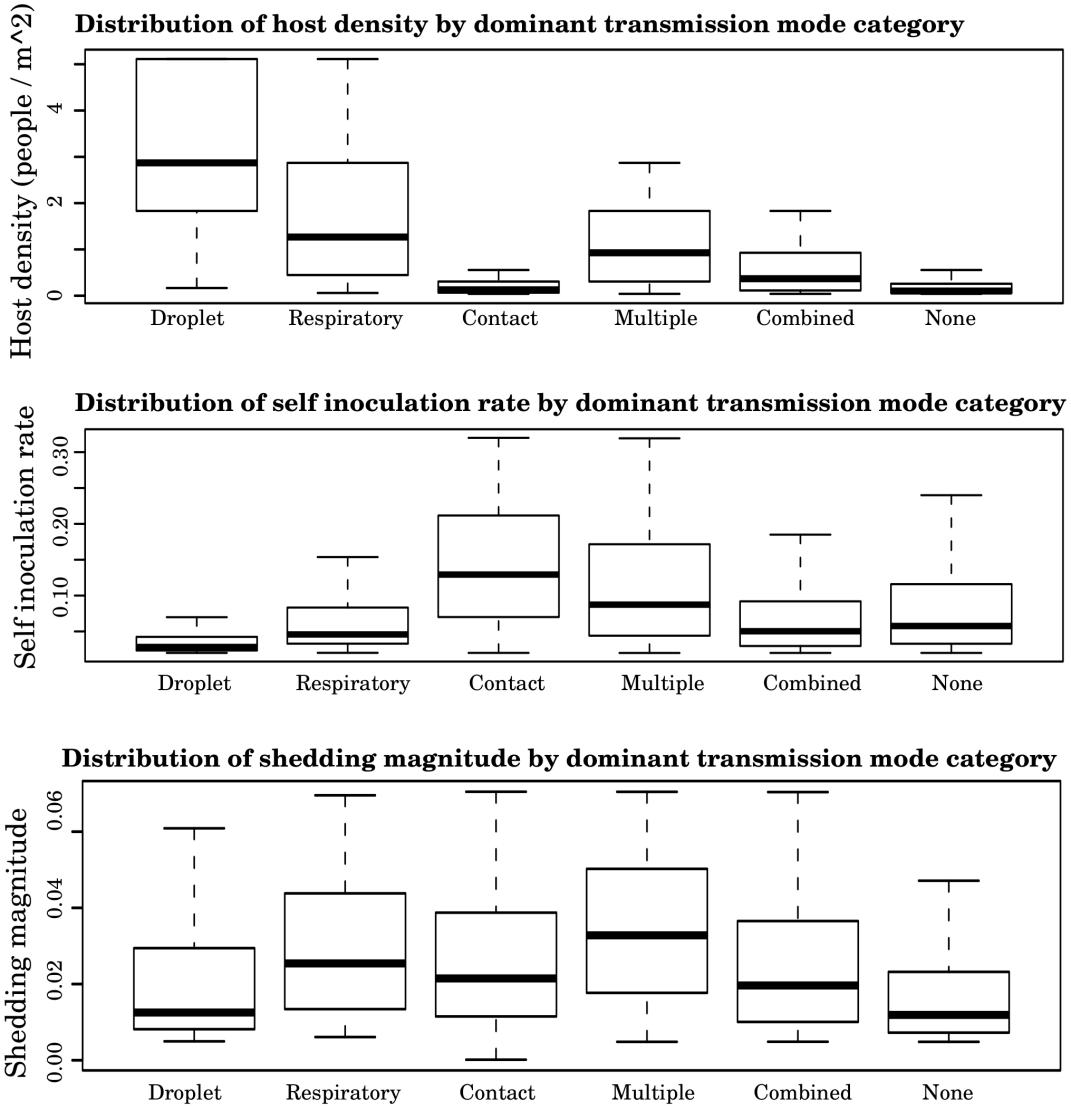
Distribution of the A) host density, B) self inoculation rate, and C) shedding magnitude parameters for different categories of transmission mode dominance. Droplet, respiratory, and contact refer to parameter sets which only yielded high transmission by these routes alone. Multiple refers to parameter sets where more than one transmission route was causing high transmission. Combined refers to parameter sets which did not contain a single dominant transmission mode, but did cause high transmission by multiple modes combined, and none refers to parameter sets which both had no dominant modes of transmission and also did not combine to cause high transmission.

Host density (*ε_density_*) shows the most striking difference in parametric distributions between the different dominant transmission mode categories (figure 4). The droplet-only category has the highest distribution of *ε_density_*, followed by the respiratory-only category. The contact-only category has a low *ε_density_* distribution, similar to the category in which there is no high transmission. Note that self inoculation rate and shedding magnitude also vary considerably between categories. Thus, features of the host, pathogen, and environment all play a role in determining transmission mode dominance. For box plots of all other parameter distributions refer to the supplemental material (figure S4, figure S5, figure S6, figure S7, figure S8, figure S9, figure S10, figure S11, figure S12, figure S13).

### Transmission mode intensity

**Figure 5:**
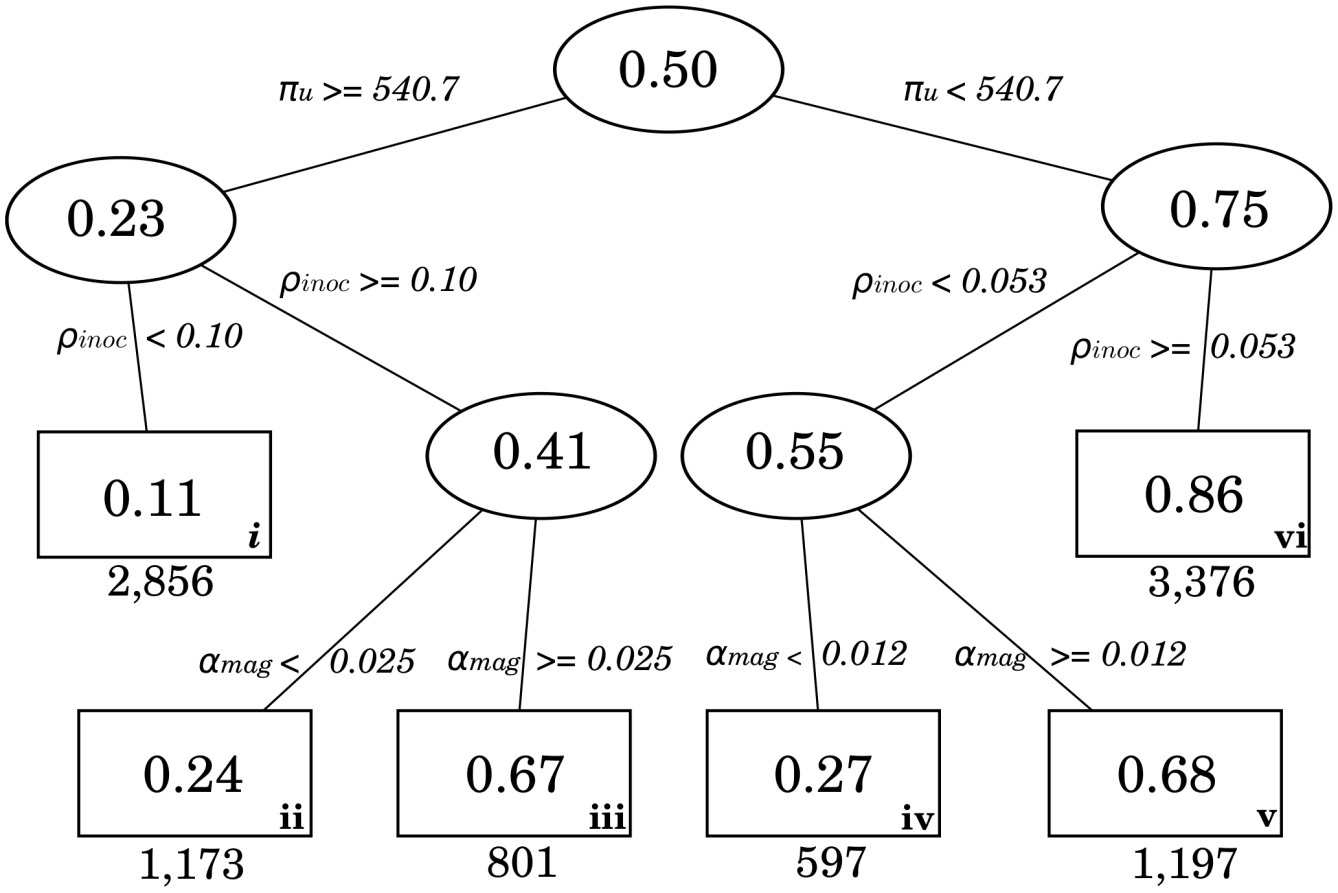
The contact-route CART diagram. Numbers in ovals and rectangles are the proportions of parameter sets have mode-specific R0>1.7 which meet the parameterization criteria shown on edges. Numbers at the bottom of each terminal node reflect the number of simulations which meet that classification criteria. Three parameters differentiate between areas of high versus low contact transmission: upper respiratory ID_50_ (*π_U_*), self inoculation *rate(ρ_inoc_)*, and shedding magnitude (*α_mag_*). Terminal nodes are labeled with lower case roman numerals for ease of reference.

To gain insight into how parameter combinations affect the intensity of each transmission mode separately, we performed CART analyses. For each route, we classified the full 10000 unit space as to whether each mode had high (mode-specific R_0_>1.7) or low transmission. The CART algorithm then grouped similar regions of this outcome by making parametric divisions. We show the CART figure of the contact route, differentiating between high and low contact mediated transmission in figure 5. The numbers given in the ovals and rectangles are the proportions of all parameter sets which have a contact-R_0_ greater than 1.7. Terminal nodes shown as rectangles are labeled with lower case roman numerals for ease of reference. The CART algorithm identified three parameters that differentiated between high and low contact mediated transmission (figure 5): upper respiratory ID_50_ (*π_U_*), self-inoculation rate (*ρ_inoc_*), and shedding magnitude. Terminal nodes iii, v, and vi all show high contact transmission with 67%, 68%, and 86% of parameter sets that have the required parameter divisions yielding high contact transmission. We also examined the strength of all other transmission routes in these terminal nodes (table 2) based on the average mode-specific R_0_ value. Because the contact and droplet routes share the same infectivity parameter, it is not surprising that while terminal node iii was largely contact-only, terminal nodes v and vi had high contact-and-droplet transmission combined in addition to high contact-only. In terminal node v, among the 818 parameter sets with high contact transmission, 475 of these also had high droplet transmission. In terminal node vi, among the 2912 parameter sets with high contact transmission, 1117 of these also had high droplet transmission. Thus these nodes represent scenarios where there is high contact-only transmission (node iii), as well as high combined contact-and-droplet transmission (nodes v and vi). The droplet-only transmission in these nodes is relatively small: 18 parameter sets in terminal node v and 5 parameter sets in terminal node vi. Terminal node iii by comparison is mainly composed of high contact-only transmission. The main parameter which differentiates between terminal node iii (high contact-only) and terminal nodes v and vi (high contact and droplet) is upper respiratory infectivity *π_U_*. The latter nodes required a more infectious agent than terminal node iii.

**Table 2:** Terminal node average mode-specific R_0_'s from the contact-route CART diagram.

	Terminal node numeral					
Mode-specific R_0_	i	ii	iii	iv	v	vi
Contact	0.72	1.34	4.84	0.60	5.87	20.76
Respiratory	0.47	0.22	0.88	0.13	0.65	0.54
Inspiratory	0.01	0.00	0.02	0.02	0.11	0.09
Droplet	0.37	0.18	0.63	1.07	4.85	3.77
Total-R_0_	1.82	1.74	6.37	2.83	11.47	25.16

NOTE. CART = Classification and Regression Tree Algorithm. Data represent the average values for domains in each terminal node of Figure 5. The average total-R0 may not be equal to the sum of all average mode-specific R_0_'s due to skewed distributions.

Turning to the plausibility of terminal node vi, two parameter constraints were required to yield high contact transmission in 86% of settings: first, a minimally constrained upper respiratory infectivity *π_U_*<540.7 (which covers 75% of the range sampled); and second, self inoculations occurring at least once every 19 minutes (*ρ_inoc_*> = 0.053/min). This *ρ_inoc_* critical value is lower (and thus more plausible) than self inoculation rates previously observed in two published studies: 1 touch every 12 minutes [26], and 1 touch every 4 minutes [27]. Thus, this combination of constraints is certainly plausible.

From similar CART analyses, the droplet mediated transmission mode intensity is differentiated by three parameters: upper respiratory ID_50_, host density, and shedding magnitude. Respiratory transmission mode intensity is differentiated by five parameters: host density, viral proportion respirable, shedding magnitude, lower respiratory ID_50_, and lung deposition fraction. To test whether tree structure is sensitive to the cut point of R_0_ = 1.7, we also construct CART figures using a cut point of 1.2. All resulting tree structures are robust, retaining similar structure, with only minor changes in the parameter values used to divide non-terminal nodes. See the supplemental material for complete discussion of the respiratory, inspiratory, and droplet CART analyses (Text S1 and figure S1, figure S2, figure S3). Also, correlation analyses in the supplemental material further describe how each parameter affects each mode of transmission.

## Discussion

This work highlights many parameters which can alter transmission mode dominance. By learning more about these transmission modes, we can better predict which modes are operating in specific scenarios. This insight can eventually help lead to definitions of 1) those factors that will enable us to predict how much transmission could take place via different modes and 2) effective interventions that can interrupt such transmissions. We have further shown that the relative importance of different influenza transmission modes may vary based on features related to the pathogen, host, or mixing venue (figure 1) that may vary based on biology, behavior and environmental factors.

For example, high host density leads to conditions where either droplet, respiratory, or multiple transmission routes simultaneously operate at a high intensity (figure 4a). The infectivity parameters of the upper and lower sites of respiratory infection are also very important in determining both absolute and relative strengths of transmission modes (in figure 5, comparing terminal node iii which is largely contact-only to terminal nodes v and vi which also have high droplet transmission and have a higher infectivity). Additionally, the self-inoculation rate was the most important behavioral parameter influencing contact-transmission (figure 4b). Thus, we have found specific features of the environment (host density), agent (infectivity) and host (susceptibility and self inoculation rate) that are important in determining transmission mode dominance.

Our results should be interpreted with the following caveats. First, the distribution of parameter sets we used does not necessarily represent the probabilistic distribution of parameter sets in all of the real world settings. Thus it would not be appropriate to say that the contact route is most important in the vast majority of real contexts. Going further, if different parameter constraints were used, the shape of the Venn diagram in figure 3 could look drastically different. However, it is likely that there would still be regions where contact, respiratory, and direct-droplet-spray dominated on their own. Second, the behavioral and movement space we examined was intentionally limited. Further elaboration of these features could induce additional differences from those we observed.

With this work, we can make several recommendations for future empirical work. The two influenza dose-response datasets study two different sites of infection using two different influenza strains. It is not clear whether all influenza strains would display a similar site-specific differential (upper versus lower respiratory tract infectivity). Empirical work examining site-specific infectivity first with one strain, and then with another would be quite valuable. This could help tease apart the relationship between innate variability of infectivity of virus strain, whether this varies by site of infection, and if this variability is similar across different strains. Another feature important to learn more about that could sway transmission dominance, is the shedding process. Specifically, examining particle size distributions and excretion rates based on type of excretion (cough, sneeze, normal breathing, speaking), examining how viral concentration varies by particle size, and quantifying how much saliva dilutes infectious nasal fluid in different types of excretions at different stages of infection would be useful.

Data uncertainty resulting from weakness of the data used for specific parameters is another motivation for future work. The surface inactivation rate, hand inactivation rate, all transfer efficiencies (as well as both infectivity parameters) are all based on datasets which contain a minimal number of data-points. If the value of these parameters lies outside of the ranges considered, these could also become quite important in altering transmission mode dominance and therefore optimal intervention choice. For this reason, more work examining these parameters would be worthwhile.

Although these results inform transmission mode dominance, this alone does not allow policy makers to make completely informed intervention decisions. Even if most transmission taking place in a given scenario is through the contact route, this does not indicate hand hygiene as the best intervention decision solely because it targets the contact route exclusively. For example, it is possible that specific features of the scenario which relate to how hand hygiene interacts with pathogens in the environment could render a hand hygiene intervention ineffective, despite the contact route operating at a high intensity if there are substantial pathogen levels in the environment thereby allowing hands to be re-contaminated as soon as future surface touching occurs. A study similar to this could be extended to include the modeling of specific interventions, and be used to characterize a specific scenario. Doing so would be part of an overall site-specific microbial risk assessment. This would involve taking into account specific features of the environment, host, and pathogen strain as well as their dynamic interactions.

Conclusions from previous work of others may differ from our work, since we considered a broad set of parameter ranges, rather than point estimates. Previous work of Atkinson and Wein (AW) [7] and Nicas and Jones (NJ) [21] differed in their assessment of the importance of contact mediated transmission. AW found it to be negligible, NJ found it to be of varying importance under different conditions, and we found it be important in many scenarios. We argue that their inferences arose from analyses constrained to highly specified regions in multidimensional parameter space, ignoring a large number of parameterization sets reflective of the heterogeneity in the host, pathogen and environment. Advocating one transmission mode specific intervention method based on inferences from such a specified scenario may often lead to ineffective decisions, under different situations. AW used a surface area to volume ratio of 3∶1m, suitable for small particles less than 6 µm [28], [29] that behave like a gas, and can possibly settle on vertical surfaces. However, larger particles will be more dominated by gravity, more likely to deposit on horizontal surfaces as indicated by table 3–5 of Hong [30]. Thus AW's surface area to volume ratio for settling sites for particles greater than 10 µm is not appropriate and will greatly dilute the pathogen surface concentration compared to pathogen air volume concentration, thus artificially diminishing the contact route compared to the respiratory and inspiratory routes. See supporting materials for additional discussion of this topic.

With this work it was our goal to highlight that there may not be one and only one dominant influenza transmission route in all settings. We are no more in the aerosol camp than the contact camp. We suggest that this is influenced by features related to the host, pathogen and environment. Depending on the specific situation one or more modes may be sufficient to cause high transmission, while in others no transmission may result. It will be important to extend this work to examine the effect of realistic interventions which aim to block or attenuate the environmental pathways included here. Additionally, similar model extensions could also address the importance of different modes of transmission in a more complex setting, such as multiple venues modeled simultaneously, that can address the network-like potential of certain venues as infection disseminators to a broader population.

## Supporting Information

Figure S1 The respiratory-route CART diagram. Numbers in ovals and rectangles are the proportions of parameter sets have mode-specific R0>1.7 which meet the parameterization criteria shown on edges. Five parameters differentiate between areas of high versus low respiratory transmission: host density (edensity), viral proportion respirable (aresp), lower respiratory ID50 (piL), shedding magnitude (amag), and lung deposition fraction (tL),. Terminal nodes are given roman numerals for ease of reference.
(4.54 MB TIF)

Figure S2 The droplet-route CART diagram. Numbers in ovals and rectangles are the proportions of parameter sets have mode-specific R0>1.7 which meet the parameterization criteria shown on edges. Three parameters differentiate between areas of high versus low respiratory transmission: host density (edensity), upper respiratory ID50 (piL), and shedding magnitude (amag). Terminal nodes are given roman numerals for ease of reference.
(4.47 MB TIF)

Figure S3 Distribution of the airborne viral inactivation rate parameter for different categories of transmission mode dominance. Droplet, respiratory, and contact refer to parameter sets which only yielded high transmission by these routes alone. Multiple refers to parameter sets where more than one transmission route was causing high transmission. Combined refers to parameter sets which did not contain a single dominant transmission mode, but did cause high transmission by multiple modes combined, and none refers to parameter sets which both had no dominant modes of transmission and also did not combine to cause high transmission.
(4.74 MB TIF)

Figure S4 Distribution of the surface viral inactivation rate parameter for different categories of transmission mode dominance. Droplet, respiratory, and contact refer to parameter sets which only yielded high transmission by these routes alone. Multiple refers to parameter sets where more than one transmission route was causing high transmission. Combined refers to parameter sets which did not contain a single dominant transmission mode, but did cause high transmission by multiple modes combined, and none refers to parameter sets which both had no dominant modes of transmission and also did not combine to cause high transmission.
(4.74 MB TIF)

Figure S5 Distribution of the skin viral inactivation rate parameter for different categories of transmission mode dominance. Droplet, respiratory, and contact refer to parameter sets which only yielded high transmission by these routes alone. Multiple refers to parameter sets where more than one transmission route was causing high transmission. Combined refers to parameter sets which did not contain a single dominant transmission mode, but did cause high transmission by multiple modes combined, and none refers to parameter sets which both had no dominant modes of transmission and also did not combine to cause high transmission.
(4.74 MB TIF)

Figure S6 Distribution of the finger-surface transfer efficiency parameter for different categories of transmission mode dominance. Droplet, respiratory, and contact refer to parameter sets which only yielded high transmission by these routes alone. Multiple refers to parameter sets where more than one transmission route was causing high transmission. Combined refers to parameter sets which did not contain a single dominant transmission mode, but did cause high transmission by multiple modes combined, and none refers to parameter sets which both had no dominant modes of transmission and also did not combine to cause high transmission.
(4.74 MB TIF)

Figure S7 Distribution of the lower respiratory infectivity parameter for different categories of transmission mode dominance. Droplet, respiratory, and contact refer to parameter sets which only yielded high transmission by these routes alone. Multiple refers to parameter sets where more than one transmission route was causing high transmission. Combined refers to parameter sets which did not contain a single dominant transmission mode, but did cause high transmission by multiple modes combined, and none refers to parameter sets which both had no dominant modes of transmission and also did not combine to cause high transmission.
(4.74 MB TIF)

Figure S8 Distribution of the upper respiratory infectivity parameter for different categories of transmission mode dominance. Droplet, respiratory, and contact refer to parameter sets which only yielded high transmission by these routes alone. Multiple refers to parameter sets where more than one transmission route was causing high transmission. Combined refers to parameter sets which did not contain a single dominant transmission mode, but did cause high transmission by multiple modes combined, and none refers to parameter sets which both had no dominant modes of transmission and also did not combine to cause high transmission.
(4.74 MB TIF)

Figure S9 Distribution of the lung deposition parameter for different categories of transmission mode dominance. Droplet, respiratory, and contact refer to parameter sets which only yielded high transmission by these routes alone. Multiple refers to parameter sets where more than one transmission route was causing high transmission. Combined refers to parameter sets which did not contain a single dominant transmission mode, but did cause high transmission by multiple modes combined, and none refers to parameter sets which both had no dominant modes of transmission and also did not combine to cause high transmission.
(4.74 MB TIF)

Figure S10 Distribution of the host movement rate parameter for different categories of transmission mode dominance. Droplet, respiratory, and contact refer to parameter sets which only yielded high transmission by these routes alone. Multiple refers to parameter sets where more than one transmission route was causing high transmission. Combined refers to parameter sets which did not contain a single dominant transmission mode, but did cause high transmission by multiple modes combined, and none refers to parameter sets which both had no dominant modes of transmission and also did not combine to cause high transmission.
(4.74 MB TIF)

Figure S11 Distribution of the viral proportion respirable parameter for different categories of transmission mode dominance. Droplet, respiratory, and contact refer to parameter sets which only yielded high transmission by these routes alone. Multiple refers to parameter sets where more than one transmission route was causing high transmission. Combined refers to parameter sets which did not contain a single dominant transmission mode, but did cause high transmission by multiple modes combined, and none refers to parameter sets which both had no dominant modes of transmission and also did not combine to cause high transmission.
(4.74 MB TIF)

Figure S12 Distribution of the surface touching rate parameter for different categories of transmission mode dominance. Droplet, respiratory, and contact refer to parameter sets which only yielded high transmission by these routes alone. Multiple refers to parameter sets where more than one transmission route was causing high transmission. Combined refers to parameter sets which did not contain a single dominant transmission mode, but did cause high transmission by multiple modes combined, and none refers to parameter sets which both had no dominant modes of transmission and also did not combine to cause high transmission.
(4.74 MB TIF)

Figure S13 Distribution of the transfer proportion from self inoculation site to target site parameter for different categories of transmission mode dominance. Droplet, respiratory, and contact refer to parameter sets which only yielded high transmission by these routes alone. Multiple refers to parameter sets where more than one transmission route was causing high transmission. Combined refers to parameter sets which did not contain a single dominant transmission mode, but did cause high transmission by multiple modes combined, and none refers to parameter sets which both had no dominant modes of transmission and also did not combine to cause high transmission.
(4.74 MB TIF)

Text S1 This document includes greater detail of the model structure, model parameterization, description of additional analyses, and a discussion comparing this work to previous relevant modeling works.
(0.24 MB DOC)
