# Supplementary figures and images for: Informing Optimal Environmental Influenza Interventions: How the Host, Agent, and Environment Alter Dominant Routes of Transmission
Source: PLoS Comput Biol. 2010 Oct 28;6(10):e1000969. doi: 10.1371/journal.pcbi.1000969 (PMC2965740; doi:10.1371/journal.pcbi.1000969)

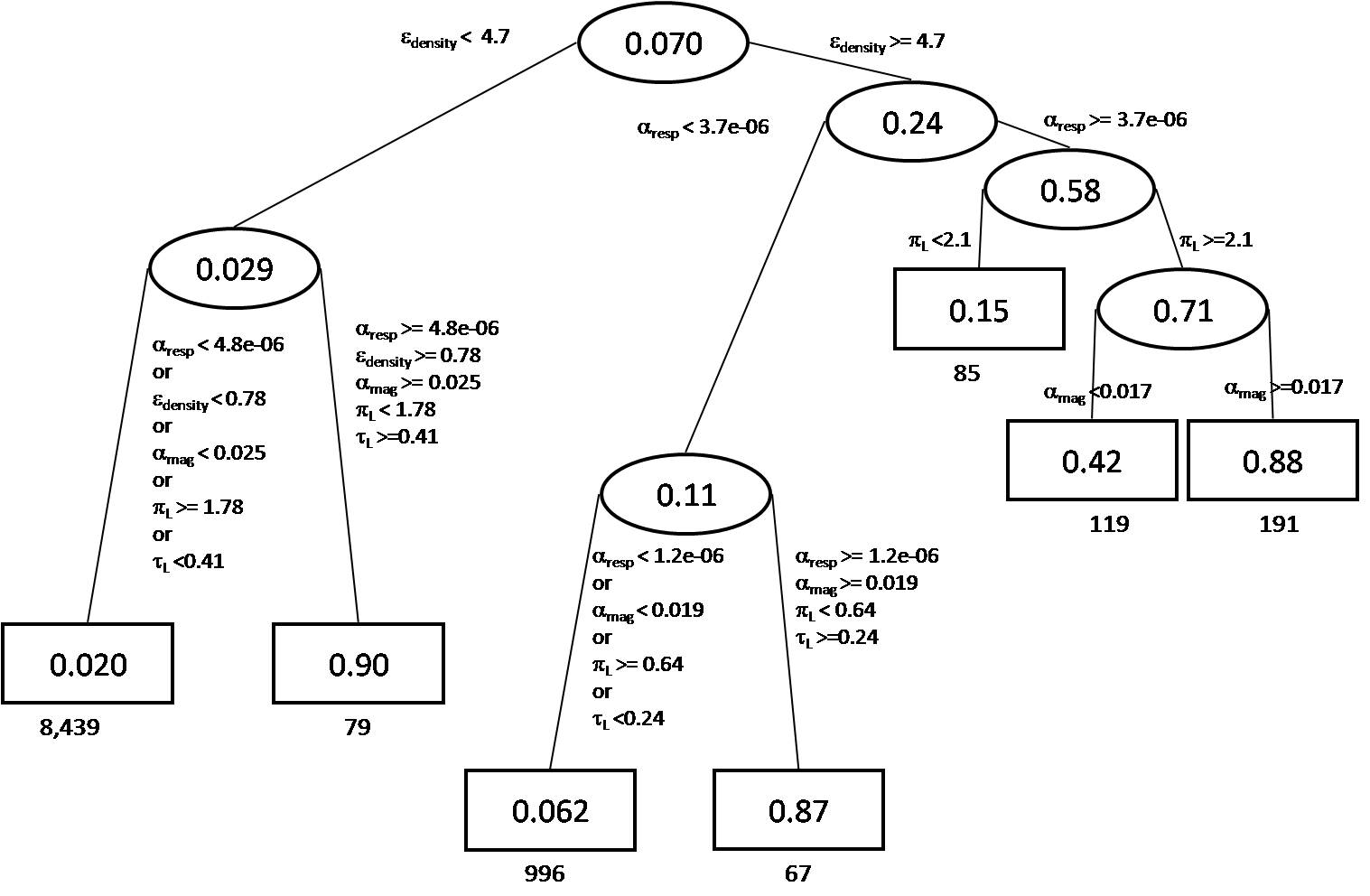

Supplement: Figure S1 — The respiratory-route CART diagram. Numbers in ovals and rectangles are the proportions of parameter sets have mode-specific R0>1.7 which meet the parameterization criteria shown on edges. Five parameters differentiate between areas of high versus low respiratory transmission: host density (edensity), viral proportion respirable (aresp), lower respiratory ID50 (piL), shedding magnitude (amag), and lung deposition fraction (tL),. Terminal nodes are given roman numerals for ease of reference. (4.54 MB TIF) [file pcbi.1000969.s001.tif]

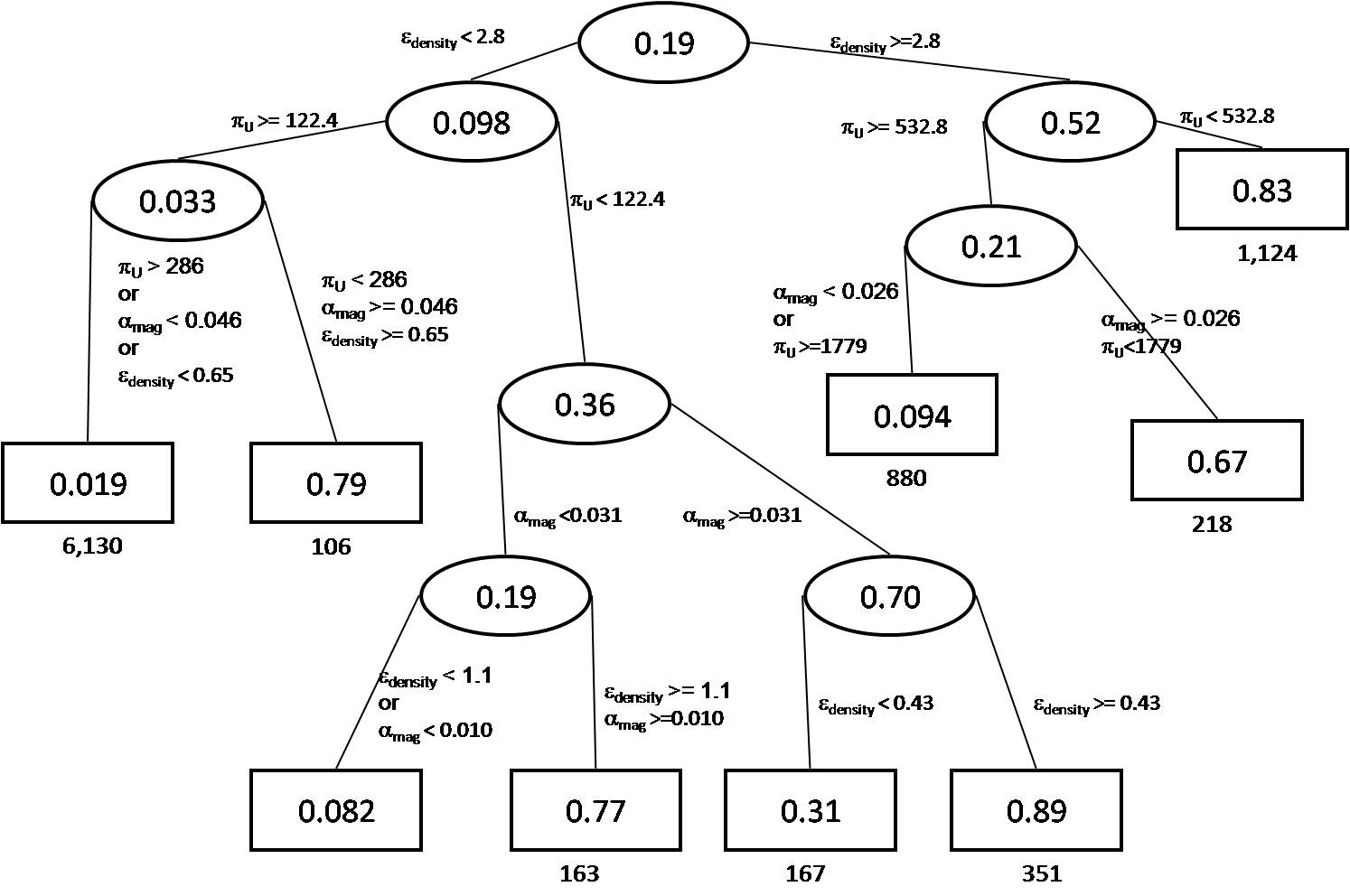

Supplement: Figure S2 — The droplet-route CART diagram. Numbers in ovals and rectangles are the proportions of parameter sets have mode-specific R0>1.7 which meet the parameterization criteria shown on edges. Three parameters differentiate between areas of high versus low respiratory transmission: host density (edensity), upper respiratory ID50 (piL), and shedding magnitude (amag). Terminal nodes are given roman numerals for ease of reference. (4.47 MB TIF) [file pcbi.1000969.s002.tif]

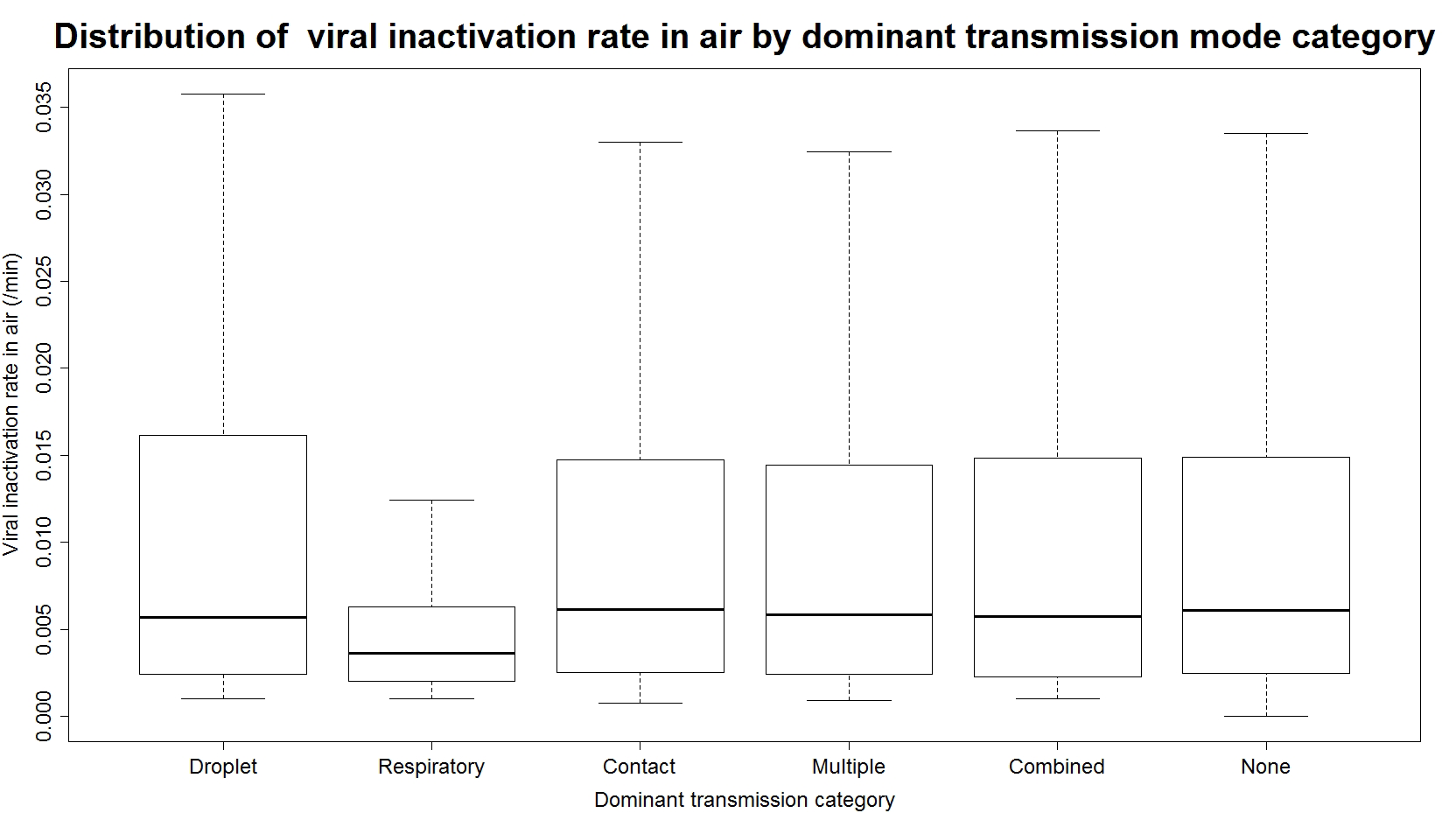

Supplement: Figure S3 — Distribution of the airborne viral inactivation rate parameter for different categories of transmission mode dominance. Droplet, respiratory, and contact refer to parameter sets which only yielded high transmission by these routes alone. Multiple refers to parameter sets where more than one transmission route was causing high transmission. Combined refers to parameter sets which did not contain a single dominant transmission mode, but did cause high transmission by multiple modes combined, and none refers to parameter sets which both had no dominant modes of transmission and also did not combine to cause high transmission. (4.74 MB TIF) [file pcbi.1000969.s003.tif]

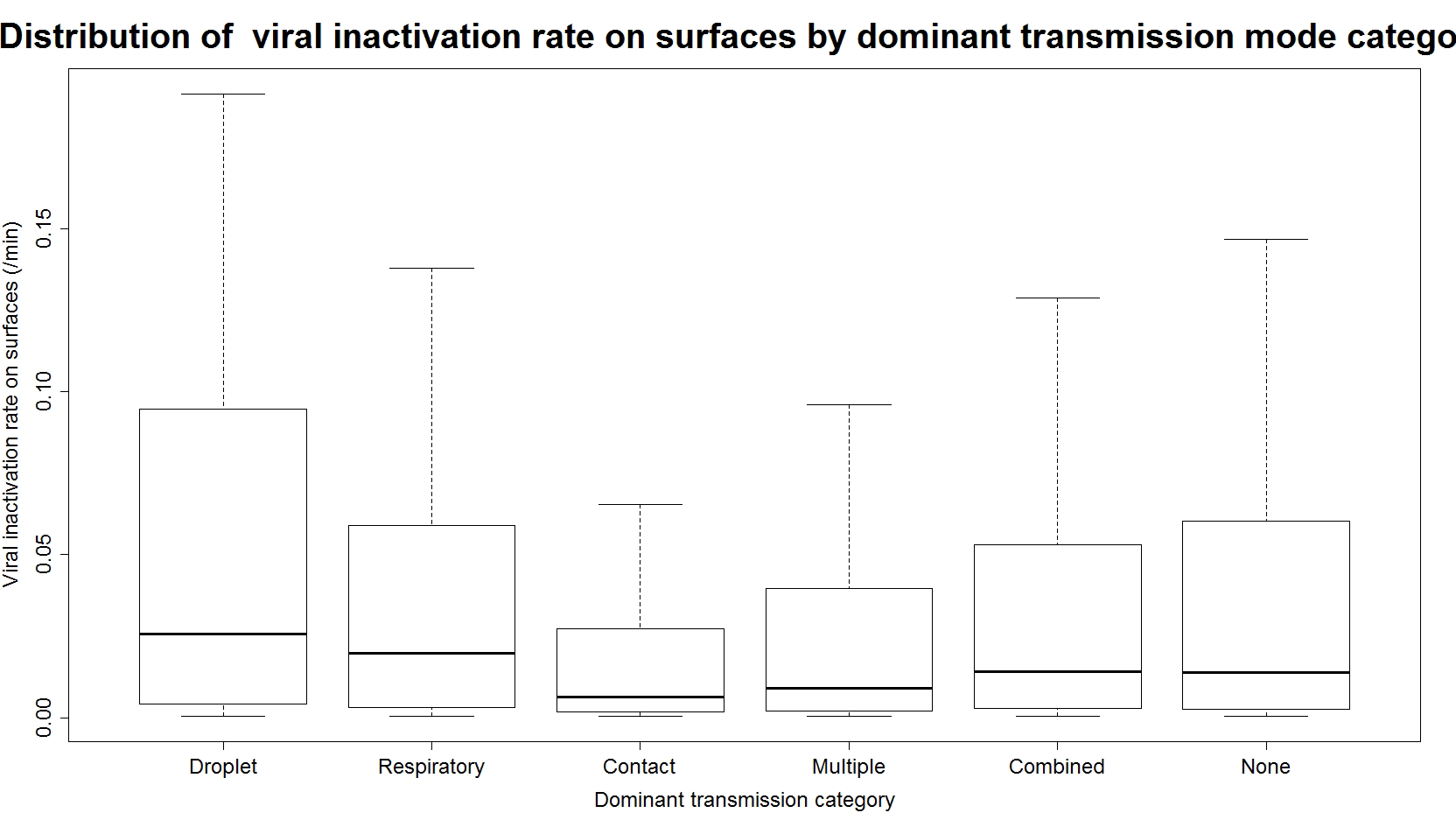

Supplement: Figure S4 — Distribution of the surface viral inactivation rate parameter for different categories of transmission mode dominance. Droplet, respiratory, and contact refer to parameter sets which only yielded high transmission by these routes alone. Multiple refers to parameter sets where more than one transmission route was causing high transmission. Combined refers to parameter sets which did not contain a single dominant transmission mode, but did cause high transmission by multiple modes combined, and none refers to parameter sets which both had no dominant modes of transmission and also did not combine to cause high transmission. (4.74 MB TIF) [file pcbi.1000969.s004.tif]

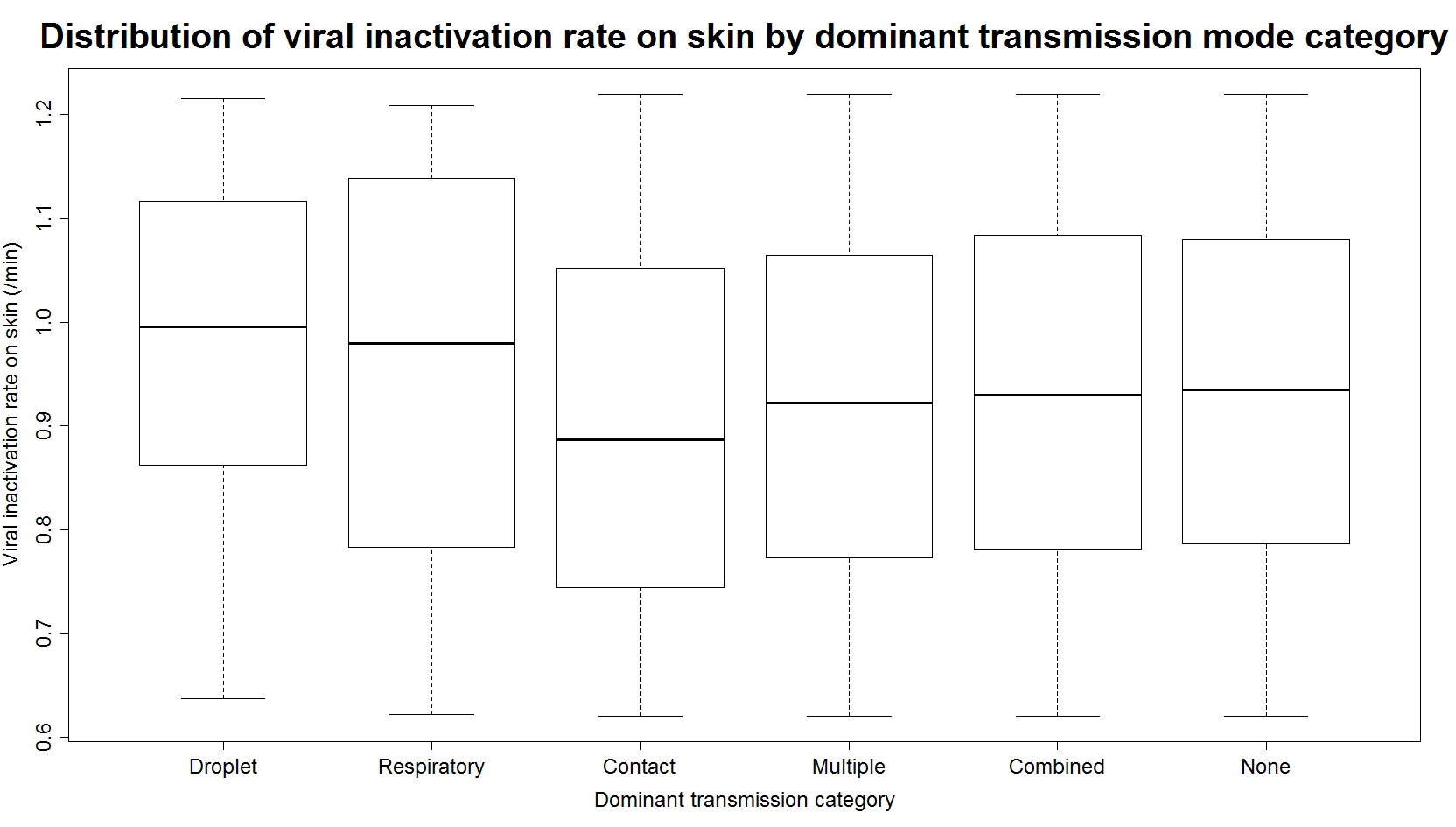

Supplement: Figure S5 — Distribution of the skin viral inactivation rate parameter for different categories of transmission mode dominance. Droplet, respiratory, and contact refer to parameter sets which only yielded high transmission by these routes alone. Multiple refers to parameter sets where more than one transmission route was causing high transmission. Combined refers to parameter sets which did not contain a single dominant transmission mode, but did cause high transmission by multiple modes combined, and none refers to parameter sets which both had no dominant modes of transmission and also did not combine to cause high transmission. (4.74 MB TIF) [file pcbi.1000969.s005.tif]

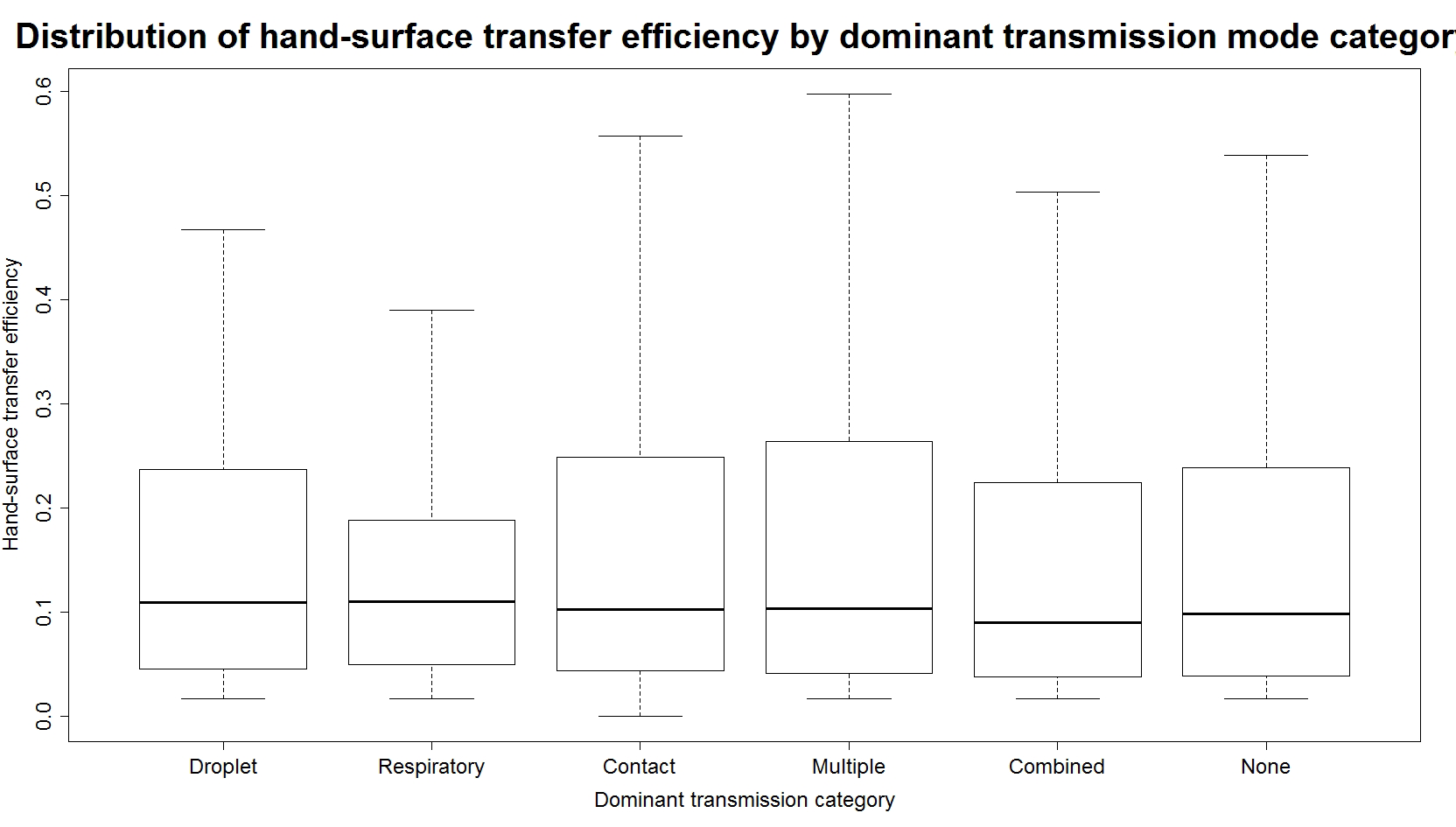

Supplement: Figure S6 — Distribution of the finger-surface transfer efficiency parameter for different categories of transmission mode dominance. Droplet, respiratory, and contact refer to parameter sets which only yielded high transmission by these routes alone. Multiple refers to parameter sets where more than one transmission route was causing high transmission. Combined refers to parameter sets which did not contain a single dominant transmission mode, but did cause high transmission by multiple modes combined, and none refers to parameter sets which both had no dominant modes of transmission and also did not combine to cause high transmission. (4.74 MB TIF) [file pcbi.1000969.s006.tif]

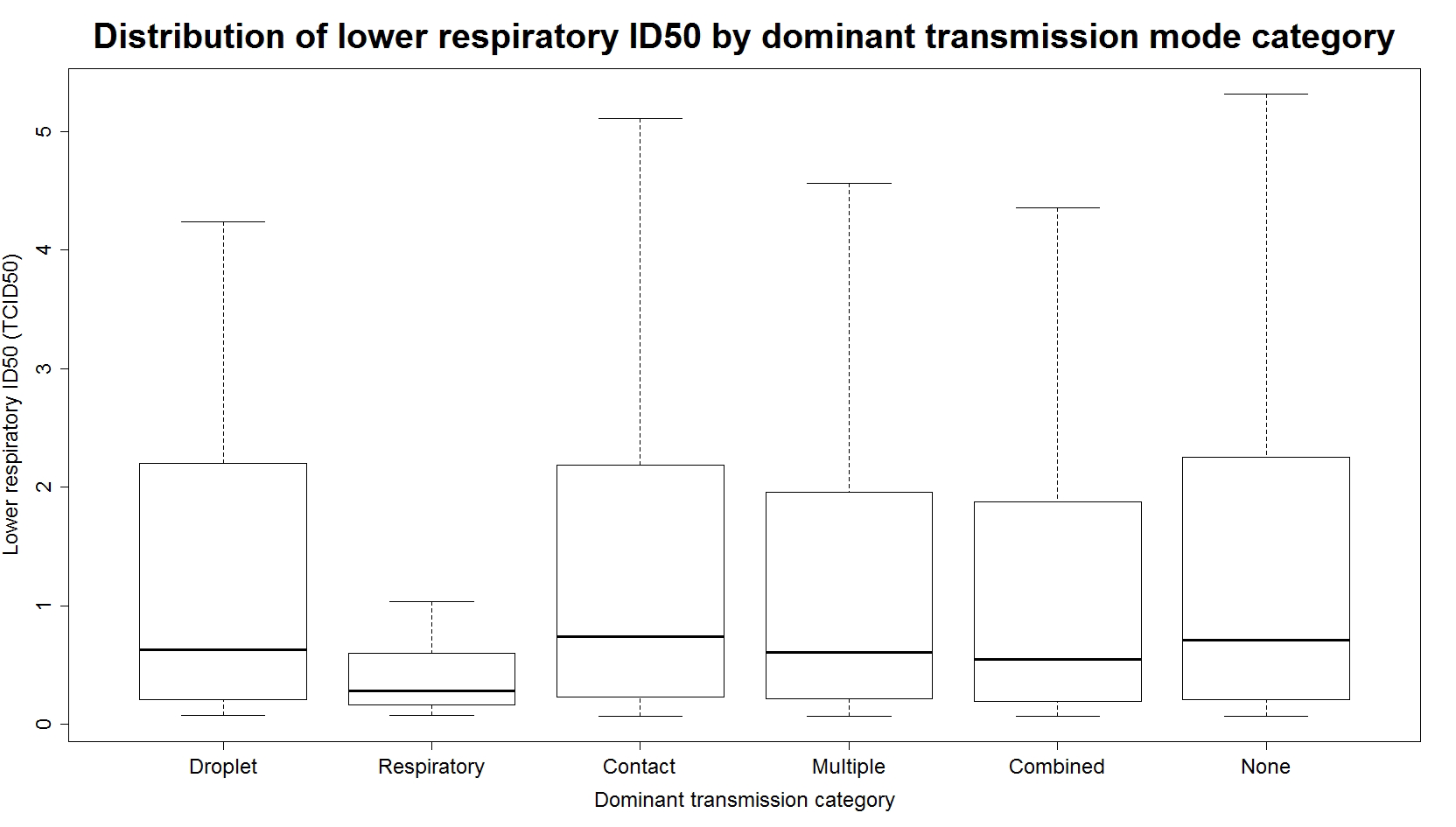

Supplement: Figure S7 — Distribution of the lower respiratory infectivity parameter for different categories of transmission mode dominance. Droplet, respiratory, and contact refer to parameter sets which only yielded high transmission by these routes alone. Multiple refers to parameter sets where more than one transmission route was causing high transmission. Combined refers to parameter sets which did not contain a single dominant transmission mode, but did cause high transmission by multiple modes combined, and none refers to parameter sets which both had no dominant modes of transmission and also did not combine to cause high transmission. (4.74 MB TIF) [file pcbi.1000969.s007.tif]

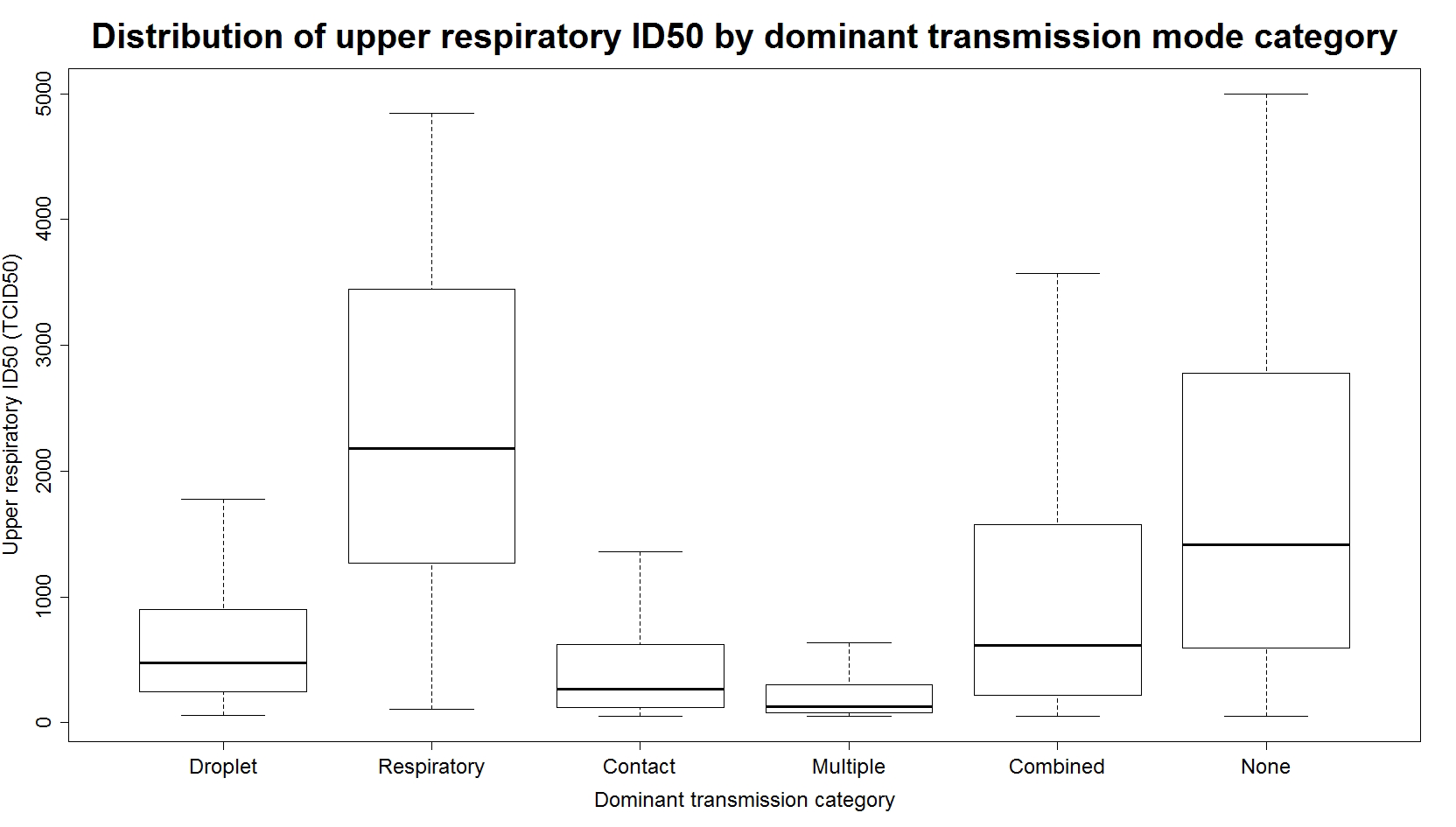

Supplement: Figure S8 — Distribution of the upper respiratory infectivity parameter for different categories of transmission mode dominance. Droplet, respiratory, and contact refer to parameter sets which only yielded high transmission by these routes alone. Multiple refers to parameter sets where more than one transmission route was causing high transmission. Combined refers to parameter sets which did not contain a single dominant transmission mode, but did cause high transmission by multiple modes combined, and none refers to parameter sets which both had no dominant modes of transmission and also did not combine to cause high transmission. (4.74 MB TIF) [file pcbi.1000969.s008.tif]

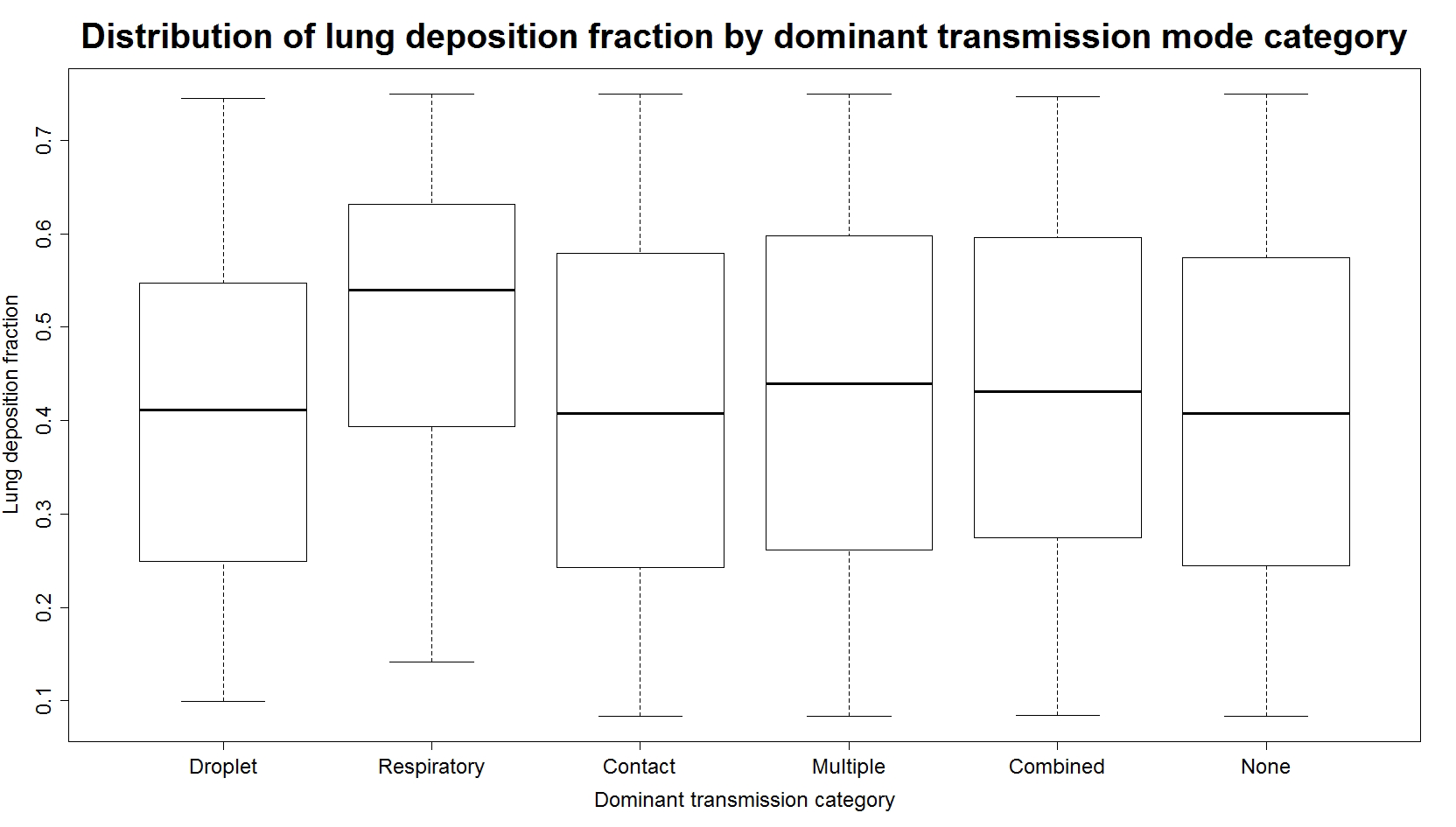

Supplement: Figure S9 — Distribution of the lung deposition parameter for different categories of transmission mode dominance. Droplet, respiratory, and contact refer to parameter sets which only yielded high transmission by these routes alone. Multiple refers to parameter sets where more than one transmission route was causing high transmission. Combined refers to parameter sets which did not contain a single dominant transmission mode, but did cause high transmission by multiple modes combined, and none refers to parameter sets which both had no dominant modes of transmission and also did not combine to cause high transmission. (4.74 MB TIF) [file pcbi.1000969.s009.tif]

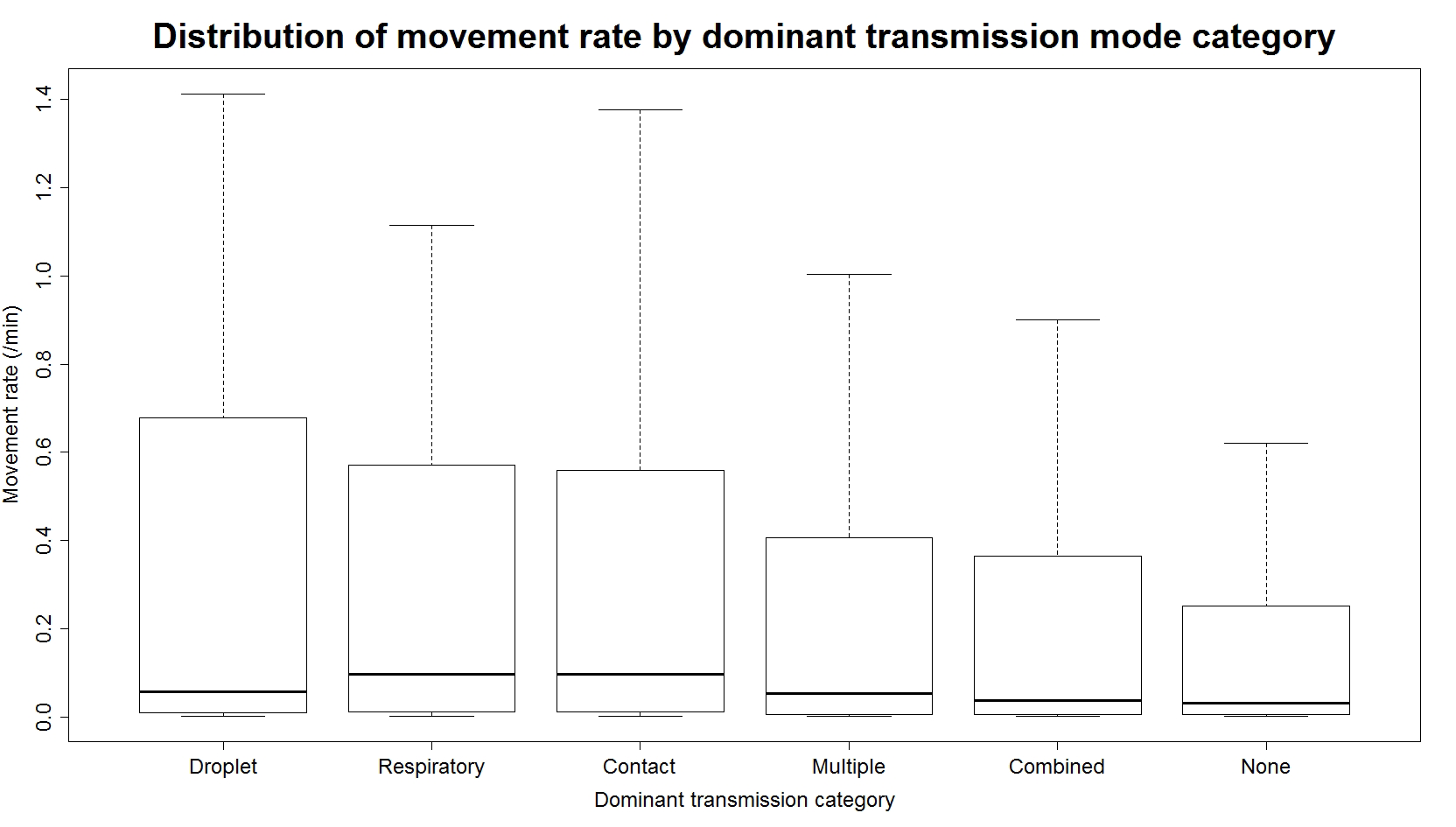

Supplement: Figure S10 — Distribution of the host movement rate parameter for different categories of transmission mode dominance. Droplet, respiratory, and contact refer to parameter sets which only yielded high transmission by these routes alone. Multiple refers to parameter sets where more than one transmission route was causing high transmission. Combined refers to parameter sets which did not contain a single dominant transmission mode, but did cause high transmission by multiple modes combined, and none refers to parameter sets which both had no dominant modes of transmission and also did not combine to cause high transmission. (4.74 MB TIF) [file pcbi.1000969.s010.tif]

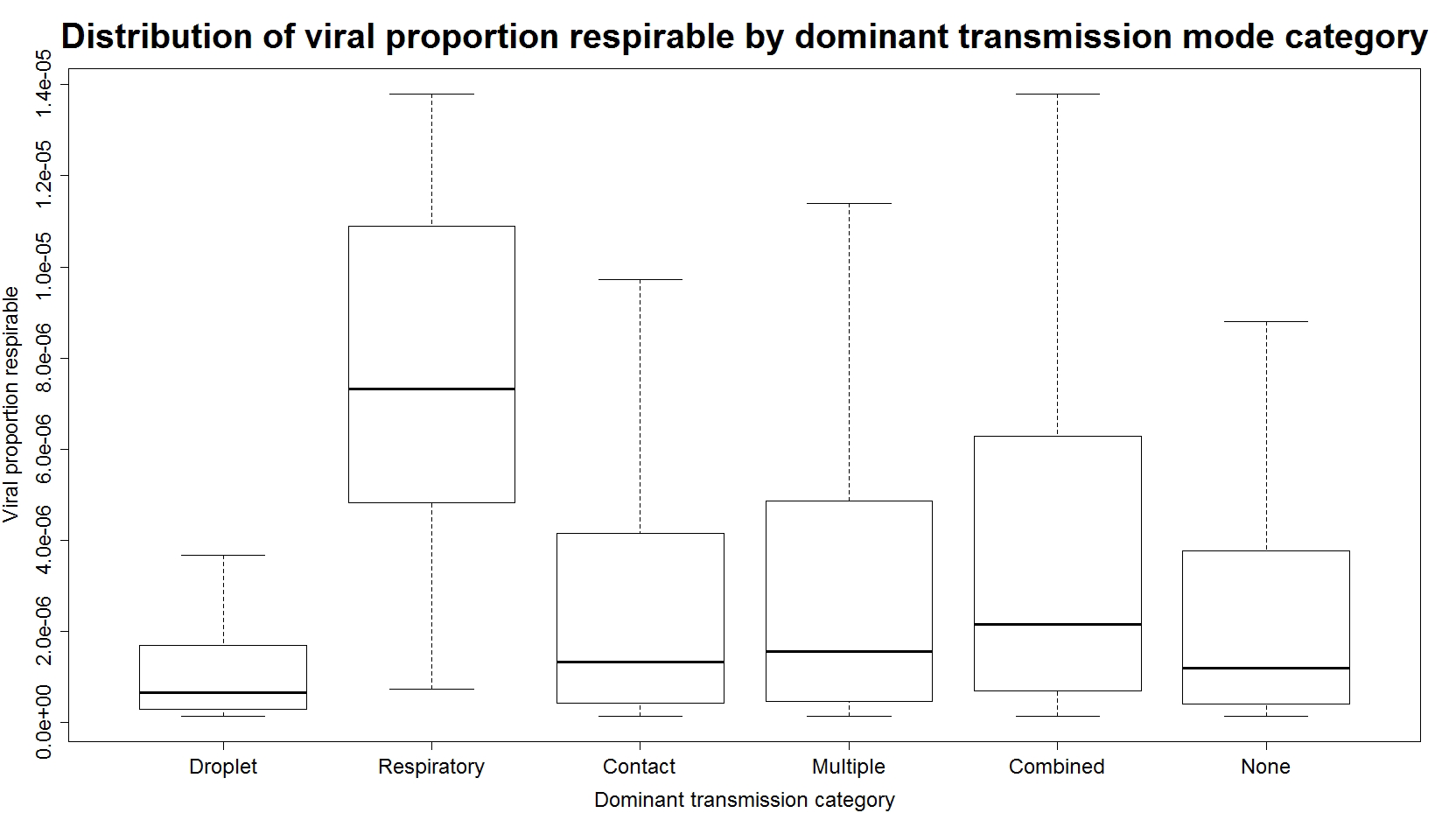

Supplement: Figure S11 — Distribution of the viral proportion respirable parameter for different categories of transmission mode dominance. Droplet, respiratory, and contact refer to parameter sets which only yielded high transmission by these routes alone. Multiple refers to parameter sets where more than one transmission route was causing high transmission. Combined refers to parameter sets which did not contain a single dominant transmission mode, but did cause high transmission by multiple modes combined, and none refers to parameter sets which both had no dominant modes of transmission and also did not combine to cause high transmission. (4.74 MB TIF) [file pcbi.1000969.s011.tif]

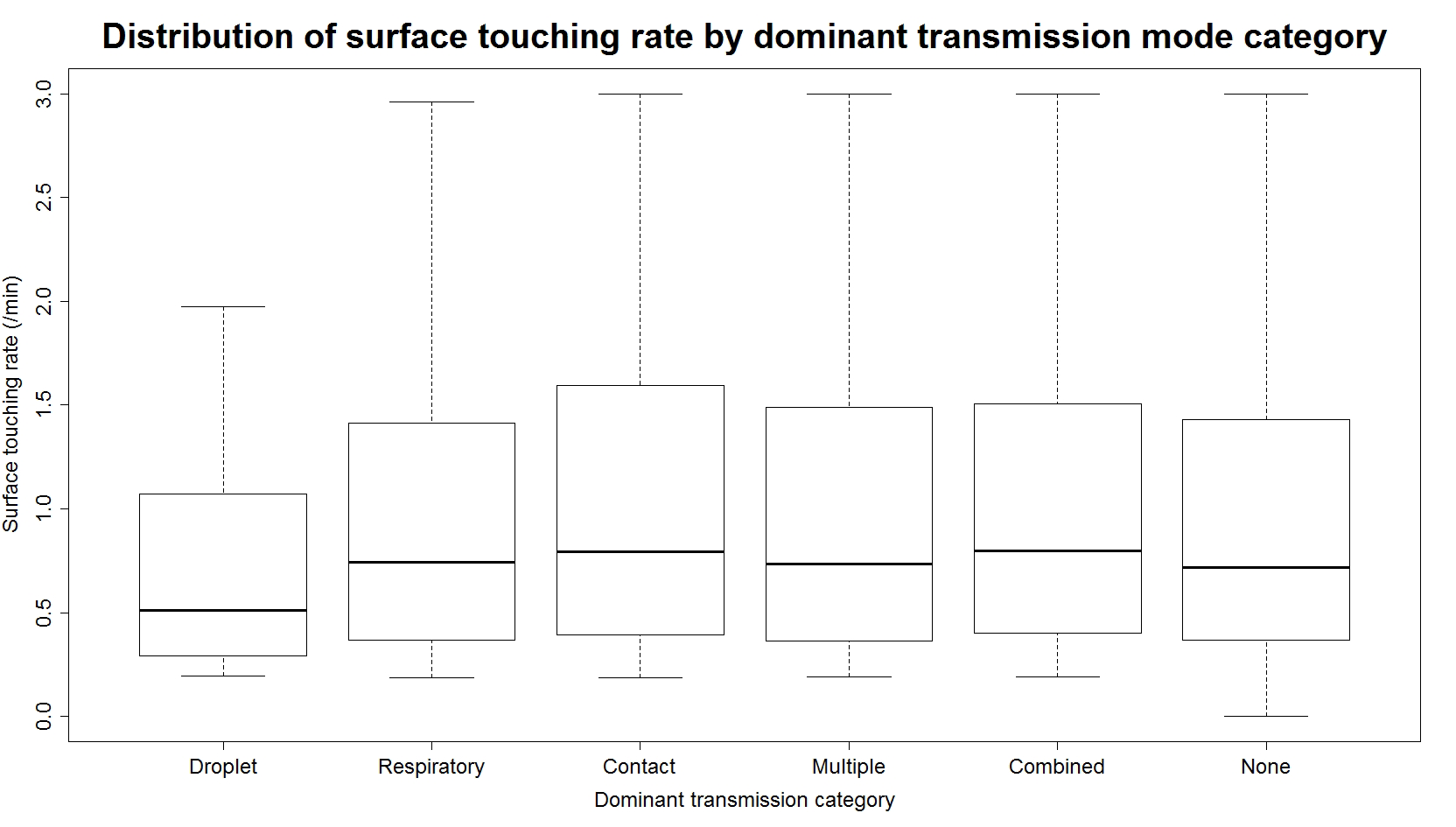

Supplement: Figure S12 — Distribution of the surface touching rate parameter for different categories of transmission mode dominance. Droplet, respiratory, and contact refer to parameter sets which only yielded high transmission by these routes alone. Multiple refers to parameter sets where more than one transmission route was causing high transmission. Combined refers to parameter sets which did not contain a single dominant transmission mode, but did cause high transmission by multiple modes combined, and none refers to parameter sets which both had no dominant modes of transmission and also did not combine to cause high transmission. (4.74 MB TIF) [file pcbi.1000969.s012.tif]

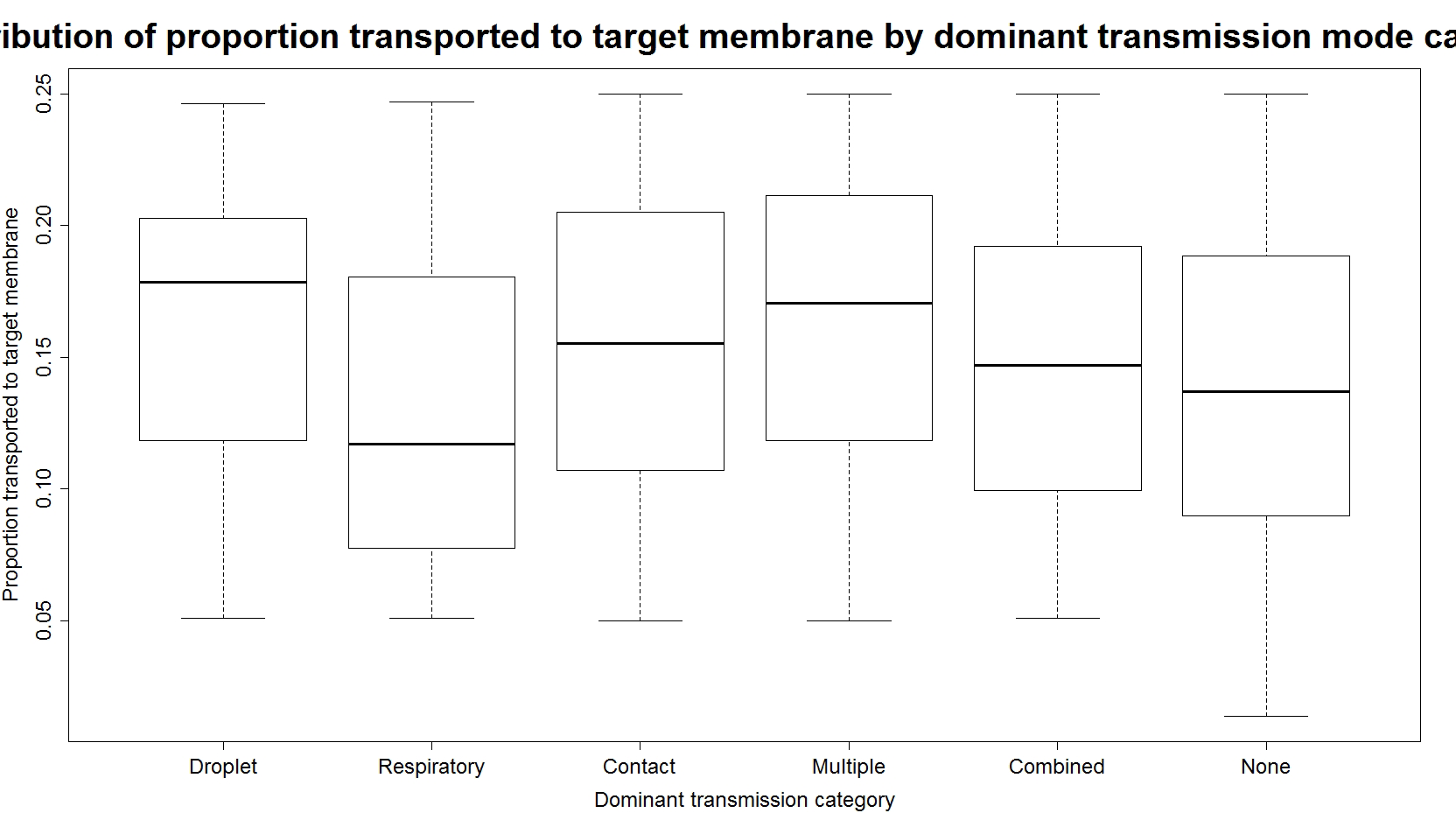

Supplement: Figure S13 — Distribution of the transfer proportion from self inoculation site to target site parameter for different categories of transmission mode dominance. Droplet, respiratory, and contact refer to parameter sets which only yielded high transmission by these routes alone. Multiple refers to parameter sets where more than one transmission route was causing high transmission. Combined refers to parameter sets which did not contain a single dominant transmission mode, but did cause high transmission by multiple modes combined, and none refers to parameter sets which both had no dominant modes of transmission and also did not combine to cause high transmission. (4.74 MB TIF) [file pcbi.1000969.s013.tif]
