# Supplementary material for: Informing Optimal Environmental Influenza Interventions: How the Host, Agent, and Environment Alter Dominant Routes of Transmission
Source: PLoS Comput Biol. 2010 Oct 28;6(10):e1000969. doi: 10.1371/journal.pcbi.1000969 (PMC2965740; doi:10.1371/journal.pcbi.1000969)
Supplement: Text S1 — This document includes greater detail of the model structure, model parameterization, description of additional analyses, and a discussion comparing this work to previous relevant modeling works. (0.24 MB DOC) [file pcbi.1000969.s014.doc]

# S1. Model structure

We explicitly model environmental influenza transmission in a venue by considering contact-mediated, respirable, inspirable, and droplet exposure.  Figure 1 gives a schematic of the processes resulting from each shedding event that leads to exposure. In our individual based model, we use continuous time to model discrete spatial units, humans, pathogens, and transmission-related events. Specific events related to transmission include shedding, viral inactivation in the environment, touching of surfaces that can contaminate hands, inhalation of air that can carry airborne pathogens, touching of one’s eyes, nose or mouth that can self-inoculate pathogens from the hands, and movement about the venue by humans. We use an event-based modeling scheme, in which we take into account the current state of the system at the current time, and based on the probabilities of all possible events, we determine what the next event will be, and when it will occur. That event is then executed at the specified time, and we then reevaluate the current state of the system to again determine which event to perform next, and when it will occur. By current state of the system, we are concerned with how many susceptible individuals, infected individuals, and influenza virus particles exist in each part of the environment. This modeling strategy is an implementation of the Gillespie algorithm [1]. The process continues until the model terminates when there are no infectious individuals still shedding and there is no remaining pathogen contamination in the environment.  The proceeding sections describe specific model components in greater detail.

**Vital dynamics**. To avoid issues related to frequency and density dependent transmission, we model a population with a constant size of susceptibles.  To do so requires an open system of people in which after each new infection event, the newly infectious individual is instantly replaced with a new susceptible.  Thus we are only allowing one infectious person to transmit.  This permits us to observe the number of new infections transmitted from one infected person over the course of their infection in the presence of a completely susceptible population of constant size—which is one definition of the basic reproductive number, R_0_[2,3].

**The Environment.** We model transmission in a single abstract venue.  Since appropriate intervention choice may vary from one venue to another, we chose to model only a single venue at a time, rather than a model of greater complexity which could consider multiple connected venues, to better understand how each transmission mode operates in different types of venues. The venue may be thought of as a lattice grid with discrete cell locations people may visit.  Each cell in the lattice has some surface area, given by its length and width (2 meters by 2 meters), and local air volume, which additionally takes into account the surface area to volume ratio (*ε_SA:V_* ).  These local fomite environments of the cell are independent of the local environments of neighboring cells, meaning that pathogens are not shared between cells unless a human picks pathogens up at one cell, moves to another, and deposits pathogens there.  At the beginning of each simulation each cell in the lattice has exactly the same characteristics and infection potential as any other.  Within each cell, we assume all pathogens are spread evenly either in air or on surface area.  Besides the local-air volume, which we assume contains only non-diffusing medium sized particles (10-100μm diameter), there is also a global air volume which the entire venue shares.  We invoke the well-mixed-room (WMR) assumption in the global air environment which contains only small particles (less than 10μm diameter), which after being excreted are assumed to have diffused instantaneously, and are evenly distributed throughout.  The global-air volume magnitude is calculated by adding together the magnitude of all the local-air volumes.  Within a given cell, when people touch a portion of the total surface area or breath a portion of the local and global volume, they are being exposed to a sample of the environment at that location with known pathogen contamination levels.

**Infection Progression.** After the initiation of infection, we consider eight stages of infection. Each stage has a specific viral concentration [4] associated with each excretion event, such that overall pathogen output starts low, rises to a peak in the second stage, and then tails off until the final stage, when it is back to the low levels of the first stage. Staged progression occurs with an expected rate of one progression per day.

**Shedding**. The infectious individual sheds pathogens over the course of their infection. The rate of pathogen excretion varies by stage of infection. Discrete shedding events, analogous to coughs, occur at a constant rate over the course of the infection, although the amount of pathogen released is not constant. Each shedding event puts out some total volume of mucous material. However only part of this volume is the potentially infectious nasal fluid (*α_mag_*). This mucous has a viral concentration which varies by stage of infection. As manuscript figure 1 indicates at the left, the shedding volume may be divided into three categories based on particle size. Some proportion of the total volume excreted has a particle size less than 10 micrometers (*α_resp_*) which we assume remains aerosolized and may potentially be respired.  We assume these <10 μm diameter particles are instantaneously homogeneously distributed throughout the venue—the WMR assumption—in the global air volume.  There is also some proportion which has a particle diameter between 10 and 100 μm (*α_insp_*).  This proportion remains aerosolized temporarily, with the potential for being inspired, until eventually settling to the local fomite environment.  This inspirable proportion is only present in the local air volume of the shedder's cell, and is evenly mixed there.  The remaining proportion, >100μm diameter, we assume either settles out from the air instantaneously to local fomite surfaces, or immediately causes droplet exposure by settling on another individual's facial membrane who is collocated with the shedder. The pathogens which settle in the local fomite environment are evenly distributed in the shedder's cell.

**Virus inactivation.** We assume a simple first order process to model the loss of viability of the virus. There are separate inactivation events and inactivation rates based on where there is virus: in the air, on fingertips, or on fomite surfaces *(μ_A_, μ_F_, μ_S_*) . In addition, virus in the air may also leave the venue due to air exchanges. Note that inactivation is not shown in figure 1 explicitly.

**Virus settling.** Virus on particles with diameter between 10 and 100μm are initially aerosolized in the cell's local air environment, but may settle to the local fomite environment at a constant rate (*ε_settle_*) as indicated in figure 1.  Rather than modeling particles with different sizes and thus different settling rates, we use only one settling rate for medium size particles, although we do vary the settling rate in our parameter sweep.  Because the settling rate of virus on particles less than 10μm in diameter is so low, we assume they all either inactivate, leave the venue due to air exchanges, or are inhaled before settling.  Thus, we don’t consider any settling of virus on smaller particles.

**Human movement.** In this model humans change cell location at a constant rate *(ε_move_*). We assume the future position to which the human moves is independent of the current position the human occupies.  We assume that time spent in transit between two spots is negligible and is ignored.  Thus in this model, humans move without respect to space, in essence teleporting to future random locations. In a sensitivity analysis, when we allowed movement to spaces near the current location (the Von-Neuman Neighborhood), all transmission mode strengths were quite similar regardless of movement type.

**Surface touching.** Humans touch surfaces at a constant rate(*ρ_touch_*). When a surface touching event occurs, pathogens may transfer to the fomite-surface, to the fingertip, to both, or to neither based on whether there are pathogens on the surface or fingertip prior to the touch event.  To determine the quantity of pathogens transferred, we consider four parameters: the surface area of the fingertip doing the touching (*A_h_*), the total surface area in a cell (*A_s_*), the transfer efficiency (the proportion to be transferred) from fingertip to surface *(τ_h-s_*), and the transfer efficiency from surface to fingertip *(τ_s-h_*). For simplicity we assume *τ_s-h_*, and *τ_h-s_* are equal and call them by one parameter *τ_s-h-s_*. Where *V_s_* and *V_f_* are the viral numbers present on the surface or hand respectively, the number of pathogens transferred to the hand is:
V_s_ * *τ_s-h-s_** A_h_ / A_s_
The number of pathogens transferred to the surface is:
 V_h_ * *\ τ_s-h-s_*

This formulation assumes that on a single local fomite surface area, all pathogens are evenly distributed. A finger only picks up pathogen from a fraction of the surface proportionate to finger size. But contamination is assumed to cover an entire surface. Thus each pick up is treated as an average pick up.

**Self-inoculation.** Humans with pathogens on their fingertips touch their eyes, nose or mouth to self-inoculate at some rate *(ρ_inoc_*). When this occurs, some proportion of the pathogens present may be transferred (τ*_h-f_*).  Then, some proportion of this may reach a target membrane to potentially initiate infection (τ*_f-t_*). This is all performed in one step considering the products of these two transfer parameters.

**Breathing.** Human breathing events occur at some rate (*ρ_breath_*). During inhalation, a specific volume of air is taken in--the tidal volume. Particles less than 10μm may be respired to the deep lung alveoli, while particles between 10 and 100 μm diameter may be inspired and deposit in the upper airway.

To determine if any pathogens are respired (lower airway deposition), we calculate the proportion of the global air volume respired in one breath as the quotient of the tidal volume and total global air volume. If any pathogens are respired, we use a lung deposition fraction (π*_L_*) to determine the proportion that actually deposit on a susceptible location in the lower respiratory tract.  If deposition does not occur we assume the virus is exhaled back to the global air volume. 

To determine if any pathogens are inspired (upper airway deposition), we calculate the proportion of the local air volume inspired in one breath as the quotient of the tidal volume and local air volume.  Here no deposition fraction or transfer efficiency is applied; rather, if any are inspired, we assume all deposit on a susceptible tissue in the upper respiratory tract.  This over estimates the dose received from inspiratory exposure which is acceptable since we find this to be low despite this overestimate.

**Infection.** Infection may result from contact-mediated, respiratory, inspiratory, or droplet exposure.  In any case, once we have determined the dose which reaches the target susceptible tissue, discussed above, we then use an exponential model of dose response, with site-specific ID_50_ values for the upper and lower respiratory regions *(π_U_ , π_L_*).  We assume the droplet, inspiratory, and contact-mediated modes target the upper respiratory tract, while respiratory exposure has a separate target tissue, the lower respiratory tract.

S2. Model Parameterization
We next discuss the model parameterization, including literature sources, variability around point estimates, and rationale where needed for deviations from literature values.

**Shedding Parameters.** When infected individuals shed, they put out some discrete number of pathogens. To determine this we consider: 1) a shedding event rate, 2) a shedding volume, 3) a pathogen concentration within the mucous; and 4) the proportions of virus transported to the respirable air, inspirable air, local fomite environment, or causing droplet exposure.

While the cough fluid volume has been estimated at 0.044 mL [5], and cough rates observed near 0.2 per minute[6] it is highly likely that both of these values vary from person, pathogen strain, and host-strain interactions. In addition, virus may also be excreted in sneezes, which would have different rates and volumes, but similar particle size distributions. We use a constant rate of 0.2 per minute, while modulating the shedding volume to account for this variability. We modify the volume with a parameter we call the shedding magnitude factor (α*_mag_*). We let this parameter vary over a wide range (0.005 to 0.075).

To determine the pathogen concentration of the fluid being shed, we consult figure 2 of Hayden [4] to create a shedding function which takes different values by stage of infection. Note the units presented in the referent figure are TCID_50_ per mL of nasal lavage fluid. It is possible to calculate the dilution used of the lavage to calculate the nasal fluid viral concentration. However, it is not apparent how to transform nasal fluid into cough fluid, as cough fluid is also partially composed of saliva. Thus, the shedding magnitude factor allows for variability in this relationship.

To determine what proportion of the total cough volume stays aerosolized and is potentially respired (α*_resp_*) we use a critical post-evaporative particle diameter of 10 μm [5]. We combine this with the cough particle size distribution data from Loudon [7].  We assume that the pathogen concentration is the same in particles greater and less than 10 μm. This indicates that 1.4E-6 of the total volume (and corresponding pathogens residing on this volume) remain aerosolized.  We use 1.4E-7 and 1.4E-5 respectively as lower and upper constraints. Here it should noted that while coughs and sneezes certainly do not have similar fluid volumes, there is evidence[5,7,8] to support that they have similar particle size distributions, indicating that the similar proportions will be going to the different environmental routes regardless of whether a cough or a sneeze is acting. Using the same data[7] and theory[5], we calculate that 0.95% of total volume is between 10 and 100 μm diameter and has the potential to be inspired (*α_insp_*) . We use 0.35% and 1.5% as the constraints around this point value.

The remaining viruses all reside on particles with diameter greater than 100 μm. We assume that droplet spray exposure only results from virus on these large particles. As a simplification, we assume the droplet spray is spread evenly over the entire cell surface area, allowing us to use ratio of the facial membrane surface area estimated at 15 cm^2^ and the total surface area in a grid cell, 4 m^2^, for each collocated individual to determine the droplet dose received for every person in that cell. The viruses on large particles that are not utilized in droplet spray exposure are assumed to settle immediately and uniformly to the local fomite environment.

**Inactivation Parameters.** We model inactivation of influenza virus in the environment as a first order decay process with rates informed by empirical literature. We use 0.006 per minute with constraints (0.01, 0.036) for inactivation in the air [9], and 0.92 per minute (0.62, 1.22) for inactivation on hands [10]. For inactivation on fomite surfaces (μ*_S_*), the literature gives values between 0.0297 and 0.12 per minute depending on the surface type [10]. To consider scenarios with different types of surfaces present and thus different surface inactivation rates, we use 0.0005 and 0.2 for lower and upper constraints. In addition to airborne inactivation, the virus may also be removed from the system via airborne ventilation. We use a moderately low air exchavnge rate of 0.3 per hour.

**Movement and Space Parameters.** Each cell within the venue is a square with side length equal to 2m.  To vary the host density (*ε_density_*) in the venue, we let the total number of venue grid cells vary from 9 to 900 which yield densities relevant to a rush hour packed subway car, in which there may be roughly 5 people per m^2^, to a more dispersed office setting, in which there may be roughly 0.05 people per m^2^.

To calculate the venue volume we use a surface area to volume ratio of 1/3m, which, if it is assumed that gravity is the dominant force of particle settling, implicitly indicates there is only one layer of surface area.  However, it may be argued that furniture may increase both horizontal surface area for potential settling and touching as well as vertical surface area.  While we assume that gravity is still the dominant force affecting >10μm diameter particle settling, to allow for relaxing of the assumption of only a single layer of surface area per unit volume, we allow the surface area to volume ratio vary using constraints of 1/1m and 1/5m. Individuals change their 2 m by 2 m grid locations at a rate of 0.05 per minute; we consider constraints of 0.00083 and 3 per minute to consider variation around this middle value.

**Surface Touching Parameters.** When a touch event occurs, we assume five fingertips each 2 cm^2^ do the touching. Informed by empirical studies of bacterial transfer between skin and surfaces [11,12], we use a middle transfer efficiency of 10% with constraints of 1.67% and 60% from fingertip to surface and vice versa.  We use plausible surface touching rate (*ρ_touch_*) of 0.75 per minute with constraints of 0.1875 and 3 per minute.  

**Self Inoculation Parameters.** When a self inoculation event occurs, we assume that only one of the fingertips does the touching. For the self inoculation rate (*ρ_inoc_*) we use 0.08 per minute with constraints of 0.02 and 0.32 per minute, informed by an observation study of hand to face contact [13,14]. Informed by empirical studies of bacterial transfer from fingertip to mouth [15], we use a transfer efficiency of 35%. The proportion of pathogens which make it from the entry orifice to the target tissue (*τ_f-t_*) is completely unknown. We use constraints of 5% to 25% yielding a middle value of 15% for this. 

**Breathing Parameters.** To model discrete breathing events, we consider a breathing rate as well as a tidal volume for each breath, rather than considering the total volume inhaled per day. We use a tidal volume of 0.6 L.  We let the breath rate (ρ*_breath_*) vary from 10 to 22 breaths per minute. This corresponds to values of adult males resting or performing light activity [16].

**Infection Parameters.** As a result of self-inoculation, inhalation, or droplet exposure, there is potential for infection to result. To calculate the dose a susceptible tissue receives during respiration, we take the ratio of the tidal volume and total air volume and multiply this by the fraction of pathogen particles which will actually deposit in the lower respiratory tract. For this lung deposition fraction (π*_L_*), we use a value between 15 and 60% [17]. Once the dose is determined we evaluate whether infection results instantaneously by using the HID_50_ of empirical dose response studies of the lower respiratory tract [18]. We convert this HID_50_ as done by others [19] to take into account the deposition fraction to the lungs for the particles sizes administered. This yields a lower HID_50_ (π*_L_* ) of 0.671 TCID_50_; we use constraints of 0.0671 and 6.71 TCID_50_. We assume a simple exponential dose response function to consider varying doses for this and all other modes.

To determine the dose received from inspiration we use the ratio of the tidal volume and the local cell air volume to determine what proportion of the total amount of virus in the local air.  We assume these particles deposit in the upper respiratory tract, and thus we use the upper respiratory HID_50_ (π*_U_* ) of 500 TCID_50_[20,21] with constraints of 50 and 5000 TCID_50_.

 During self inoculation events the dose received is calculated as described previously in the self inoculation parameterization section. This yields the magnitude of virus which eventually gets from entry orifice to target membrane. This dose is evaluated using the upper HID_50_ (π*_U_* ) as it is assumed that the site of infection is in the upper respiratory tract.

At each shedding event, collocated individuals may receive droplet exposure.  The dose is determined by taking into account the facial surface area of each collocated individual divided by the total surface area of the cell.  In addition, we use the same transfer proportion from entry orifice to target membrane as in the contact mediated mode to diminish the dose received. To evaluate whether infection results we again use upper HID_50_ (π*_U_* ), as it is assumed the site of infection is in the upper respiratory tract.

# S3. Means of each category from Venn diagram

| Parametric means in different transmission mode dominance categories. | | | | | | | |
| --- | --- | --- | --- | --- | --- | --- | --- |
|  | Global Mean | Combined below | Contact only | Resp. only | Droplet only | Only combined above | Multiple modes above |
| N | 10000 | 4188 | 3079 | 121 | 66 | 577 | 1969 |
| inactivation in air | 0.00976 | 0.0099 | 0.00981 | 0.00573 | 0.0108 | 0.0097 | 0.0096 |
| inactivation on surfaces | 0.0333 | 0.04 | 0.0246 | 0.0389 | 0.0544 | 0.0373 | 0.0305 |
| inactivation on hands | 0.92 | 0.9312 | 0.8987 | 0.9605 | 0.9816 | 0.9328 | 0.9210 |
| proportion transported to target membrane | 0.15008 | 0.1405 | 0.1552 | 0.1318 | 0.1598 | 0.1473 | 0.1641 |
| lung deposition fraction | 0.4167 | 0.4115 | 0.4106 | 0.4984 | 0.4081 | 0.4321 | 0.4283 |
| lower-respiratory ID50 | 1.4427 | 1.4982 | 1.4847 | 0.4536 | 1.4914 | 1.3341 | 1.3450 |
| upper-respiratory ID50 | 1075.05 | 1777.89 | 549.88 | 2362.3 | 776.00 | 1064.55 | 335.84 |
| shedding magnitude | 0.02585 | 0.0189 | 0.028229 | 0.0304 | 0.0205 | 0.0263 | 0.0367 |
| viral proportion respirable | 2.97E-06 | 2.70E-06 | 2.88E-06 | 7.60E-06 | 1.44E-06 | 3.80E-06 | 3.19E-06 |
| self inoculation rate | 0.1082 | 0.0857 | 0.1438 | 0.067 | 0.037 | 0.0765 | 0.1147 |
| surface touch rate | 1.0144 | 0.9842 | 1.0536 | 0.9812 | 0.7813 | 1.0304 | 1.0214 |
| surface area to volume ratio | 3.0009 | 3.0474 | 3.0050 | 2.485 | 3.1847 | 2.9866 | 2.9254 |
| host density | 0.6314 | 0.4977 | 0.5472 | 2.434 | 7.904 | 0.8475 | 1.8566 |

# S4. Description of CART figures

**Respiratory CART.** As indicated by CART analysis five parameters were important in differentiating between regions of high (R_0_>1.7) and low respiratory transmission (R_0_<1.7): host density (*ε_density_*), viral proportion respirable (α*_resp_*), shedding magnitude (α*_mag_*), lower ID_50_ (*π_L_*), and lung deposition fraction (τ*_L_*).

In terminal node ii the respiratory mode was capable of causing pandemic transmission in 90% of the observed scenarios; requirements included at least 4.8E-6 of all pathogens being excreted to particles less than 10 micrometers in diameter, a host density between 0.78 and 4.7 people per m^2^, a shedding magnitude greater than 0.025, lower ID_50_ less than 1.78 TCID_50_, and a lung deposition fraction greater than 41%. When host density was less than 4.7 people per m^2^ and at least one of these other conditions was not met, only 2% of scenarios showed pandemic level transmission caused by the respiratory route by itself. In addition to terminal nodes ii, terminal nodes iv and vii also indicated high respiratory transmission. Of the three nodes in which greater than 87% of parameter sets yielded high transmission, note that in two of these it was not necessary to have a very infectious strain (high *π_L_*). In all of these high respiratory transmission terminal nodes, the contact mode was also high in roughly 80% of parameter sets which gave rise to each node (Section S6). This indicates that these nodes did not pinpoint any high respiratory-only contexts.

We have shown that similar contexts in terms of high respiratory transmission may be achieved as a result of a tradeoff between the parameters in figure 2—lower values of one which dampen transmission, may be counteracted by higher values of others. For example, in terminal node ii, moderate ε_density_ (between 0.78 and 4.7 people per m^2^) and weak π_L_ (<1.78) are counteracted by high α_resp_ (>4.8E-4) and high τ_L_ (>41%). In terminal node iv the situation is reversed with a more moderate α_resp_ (between 1.2E-6 and 3.7E-6) and low τ_L_ (>24%) being counteracted by high ε_density_ (>4.7 people per m^2^) and stronger π_L_ (<0.64 TCID_50_). It is also interesting to consider what respiratory parameters were not present in the CART figure: viral inactivation in the air (*μ_A_*), breath rate (*ρ_breath_*), and surface area to volume ratio (ε*_SA:V_*). These parameters were still influential in causing respiratory transmission, as indicated by correlation analysis (Section S7), but they do not differentiate between high and low transmission as much as others.

**Contact CART** To differentiate between high and low transmission intensity for the contact mediated transmission mode, three parameters were indicated: upper ID_50_ (*π_U_*), self-inoculation rate (*ρ_inoc_*), and shedding magnitude (*α_mag_*). Terminal nodes iii, v, and vi all show high contact transmission. Terminal node iii was largely contact-only producing high transmission, while the droplet mode was causing high transmission in terminal node v and vi in about 41% and 31% of these parameter sets respectively. As shown by terminal node vi, given a *π_U_* in the more infectious 3/4 of the range considered (*π_U_* < 540.7) and self inoculations occurring at least once every 19 minutes, 86% of scenarios resulted in high contact transmission. This *ρ_inoc_* critical value is lower than self inoculation rates previously observed: 1 touch every 12 minutes [13], and 1 touch every 4 minutes [14]. Thus it is likely a realistic parameter region. Note that viral inactivation rate on hands (*μ_H_*) and viral inactivation rate on surfaces (*μ_S_*) are not present in figure 3. Thus, in the parameter range we considered, these parameters did not greatly differentiate between high and low transmission settings. On hands, the range corresponded to an expected pathogen lifetime between 48 seconds and 96 seconds. On surfaces, the range was larger; the expected lifetime was between 5 minutes and 33 hours with a median expected value of 100 minutes. This helps explain the importance of *ρ_inoc_* in figure 3 rather than *ρ_touch_*. Given an environmental persistence on surfaces of roughly an hour, the bottleneck in the exposure cycle occurs on the fingertip before self-inoculation, rather than in the environment before getting picked up. The self inoculation rate must be high enough to overcome the high inactivation rate on the skin. Given a *π_U_* in the less infectious half of the range considered, and ρ*_inoc_* less than 1 touch every 10 minutes, low transmission occurs in 89% of parameter sets.

**Droplet CART.** The intensity of the droplet mediated transmission mode is differentiated by three parameters: upper ID_50_ (*π_U_*), host density (*ε_density_*), and shedding magnitude (*α_mag_*). Like the respiratory mode, these parameters may compensate for one another to yield high droplet transmission. For example in terminal node ix, under high host density( ε_density_>2.8 people/m^2^) and *π_U_* < 532.8, 83% of scenarios resulted in high droplet transmission, but with a less infectious agent (*π_U_* > 532.8) only 21% of scenarios indicated high droplet transmission. Low host density conditions (ε_density_<2.8 people/m^2^) may be overcome to achieve high droplet given other compensating parameter values, as in the case of terminal node ii; here 79% of scenarios result in high droplet transmission given a very infectious agent (*π_U_* < 286), moderate shedding magnitude (*α_mag_> 0.046*) and excluding very low host densities (0.65 < ε_density_ < 2.8). Thus at low host densities, a high infectivity is required for substantial droplet transmission. High droplet transmission is indicated in terminal nodes ii, iv, vi, viii, and ix. In all of these terminal nodes, there was high contact transmission in at least 80% of parameter sets. In terminal node viii, there was high respiratory transmission in about 35% of this node.

**Inspiratory CART.** Strength of inspiratory transmission is differentiated by host density (*ε_density_*), upper ID_50_ (*π_U_*), shedding magnitude (*α_mag_*), and surface area to volume ratio (*ε_SA:V_*). Relatively high host density, infectivity, shedding magnitude, and surface area to volume ratio are all required to cause substantial inspiratory transmission. These conditions are only satisfied by one node in the tree representing 11 parameter sets. Assuming our parameter constraints are reasonable, inspiratory transmission may be the least important influenza mode, as it requires the most extreme (and potentially unlikely) values of these four parameters jointly. This node also had high transmission by the contact and droplet routes.

# S5. Comparison to previous models

To compare our modeling approach and results to previous models, we summarize major differences in model parameterization and model structure. The model of Atkinson and Wein (AW) has only one major parametric difference. They use a surface area to volume ratio of 3:1m. This ratio is generally used to inform particle settling within a given room volume for settling of extremely fine particles in gases. It is unrealistic however for larger particles, where settling is dominated by gravity, making it unlikely that they would settle on non-horizontal surface area. Given a room height of 3m with one layer of horizontal surface area to settle to, the appropriate ratio would be 1:3 m, the inverse of what AW used. It is possible that are multiple surface area layers, or that particles may settle to vertical surfaces; these conditions would result in a ratio closer to AW’s value, but would still unlikely reach their value. For these reasons we considered values from 1:1m to 1:5m.

Structurally, important differences in AW’s approach relates to assumptions of behavior of individuals in their model. First, the symptomatic shedder does not leave their private room. Second, the symptomatic shedder is only visited by one other person. These two features only allow the possibility of the contact mode infecting the single care-giver, while the airborne routes are allowed to continue to operate on all individuals, as viruses in the air may diffuse from the shedder’s private room to other areas.

The parametric surface area to volume ratio value and structured human movement in concert greatly magnify the potential of the airborne routes compared to others from AW. Thus, it is not surprising that AW’s solution space was not observed in our results. For example, the inspiratory route in AW, accounted for 36% of all transmissions, while the maximum observed in our 10,000 unit set was only 12.5%. The surface area to volume ratio as well as the strict social isolation imparted in AW can explain this discrepancy. The prior inflates both airborne routes relative to the contact route, while the latter further weakens both the droplet and contact routes, both of which require close proximity. Because of these differences our relative contact, droplet respiratory, and inspiratory transmission vector is different from AW. However, the ratio of respiratory to inspiratory transmissions should not have similar differences resulting from different implementations. In fact 600 parameter sets exist in our results fitting AW’s respiratory to inspiratory ratio solution space.

All parameter values used in Nicas and Jones (NJ) fell within the ranges of parameter values included in our sample. However, like AW, NJ was structurally modeling a specific scenario: the transmission resulting from a caregiver visiting a symptomatic individual’s room for 15 minutes. The major difference here is that proximity is maintained between the shedder and susceptible for the entire modeled period. This over emphasizes transmissions which require close proximity: the droplet and contact mediated routes.

We were able to find each of the solution spaces NJ summarized. Each space accounted for less than 200 (2%) of our full 10,000 unit space. We summarized the parameter distributions that gave rise to 3 of NJ’s solution spaces and compared the parameter distributions in each of these to the parameter distribution of the vectors in our sample not part of each subsection. Each parameterization presented in NJ is only a small subset of all possible parameterizations which could produce such relative transmission vectors; conceptually, the same is true of AW—there are many parameterizations which could have produced their relative transmission vector—we just did not consider any of these to be in a realistic region of parameter space.

As both NJ and AW defined their outcomes in terms of a relative transmission vector, additionally note that one solution space may cover a broad range of total transmission intensities, from very low to very high total force of infection. For example, a solution space for AW or NJ that suggests dominance in air and droplet may be under the constraint that total-R0 < 1 or total-R0 > 3. Each of these situations has major implications with respect to intervention choices. Decisions on whether to intervene may vary based on total force of infection, and threshold values of each mode-specific absolute intensity may also alter intervention decisions.

There were three key differences between our approach and that of both AW and NJ. First, AW used a surface area to volume ratio of 3:1m, which is suitable for small particles less than 6 μm [22,23]. These small particles will behave more like a gas, with settling possible on vertical surfaces, while larger particles will be more dominated by gravity, more likely to deposit on horizontal surfaces as indicated by Table 3-5 of Hong[24]. Thus AW’s surface area to volume ratio for settling sites for particles greater than 10 μm is not appropriate and will greatly dilute the pathogen surface concentration compared to pathogen air volume concentration, thus artificially diminishing the contact route compared to the respiratory and inspiratory routes. This could be one reason why AW found the contact mode to be negligible as well as why we were not able to observe AW’s full solution space in our results.

Second, NJ did not model the transfer which occurs between the pathogen being transferred to the eyes/nose/mouth to the target tissue in the upper airway. NJ acknowledged this, but chose to ignore it as there was no relevant data available. AW on the other hand, while not explicitly modeling this, used a composite contact transmission parameter, which when dissected is consistent with using an additional parameter to decrease the number of pathogens being transferred to the target tissue from the eyes/nose/mouth. Our parameter sweep encompasses the region which AW pinpointed, while it does not include NJ, as NJ implicitly assumed that 100% was transferred.

Third, both AW and NJ assumed a specific contact schedule between the infectious person and at least one susceptible. This further specified a context, as defined by a specific script for interaction. This contrasts with our random movement approach, which while not necessarily modeling any specific interaction, may be the most generalizable to all non-scripted interactions. We allowed close proximity interactions to result from changes in host density and human movement in the venue, rather than scripting specific interactions. NJ required close proximity while AW only allowed one caregiver to have close proximity for a short time period and did not allow proximity otherwise. This may be the most influential factor explaining why similar inferences were not drawn from our work compared to AW and NJ.

# S6. Tabular description of terminal nodes

| Respiratory CART Tree |  |  |  |  |  |  |  |
| --- | --- | --- | --- | --- | --- | --- | --- |
| Terminal Node Number | i | ii | iii | iv | v | vi | vii |
| Contact R_0_ | 7.55 | 18.52 | 12.84 | 27.28 | 11.59 | 6.16 | 19.69 |
| Respiratory R_0_ | 0.19 | 5.38 | 0.47 | 3.87 | 1.07 | 2.49 | 9.48 |
| Inspiratory R_0_ | 0.02 | 0.09 | 0.20 | 0.37 | 0.17 | 0.08 | 0.29 |
| Droplet-spray R_0_ | 0.85 | 2.64 | 8.17 | 18.65 | 6.65 | 3.52 | 15.99 |
| Total R_0_ | 8.61 | 26.63 | 21.68 | 50.18 | 19.48 | 18.54 | 45.45 |

| Inspiratory CART Tree |  |  |  |  |
| --- | --- | --- | --- | --- |
| Terminal Node Number | i | ii | iii | |
| Contact R_0_ | 8.06 | 13.49 | 99.08 |  |
| Respiratory R_0_ | 0.32 | 2.77 | 1.89 |  |
| Inspiratory R_0_ | 0.03 | 0.24 | 4.01 |  |
| Droplet-spray R_0_ | 1.21 | 11.90 | 99.16 |  |
| Total R_0_ | 9.70 | 28.41 | 204.14 |  |

| Droplet-Spray CART Tree |  |  |  |  |  |  |  |  |  |
| --- | --- | --- | --- | --- | --- | --- | --- | --- | --- |
| Terminal Node Number | i | ii | iii | iv | v | vi | vii | viii | ix |
| Contact R_0_ | 3.27 | 32.88 | 10.35 | 21.91 | 25.78 | 45.25 | 1.48 | 6.10 | 23.81 |
| Respiratory R_0_ | 0.17 | 0.76 | 0.08 | 0.32 | 0.18 | 0.50 | 1.29 | 2.59 | 1.64 |
| Inspiratory R_0_ | 0.01 | 0.07 | 0.02 | 0.07 | 0.04 | 0.12 | 0.02 | 0.08 | 0.29 |
| Droplet-spray R_0_ | 0.27 | 3.41 | 0.76 | 3.05 | 1.42 | 5.24 | 0.72 | 3.41 | 12.79 |
| Total R_0_ | 3.71 | 37.11 | 11.20 | 25.35 | 27.41 | 51.11 | 4.34 | 12.19 | 38.53 |

# S7. Correlation analysis

This correlation analysis was performed mainly to show that the model was behaving as we would expect; e.g.. features that were programmed to affect only the contact route of transmission were only associated with it.

| Respiration Infection |  |  |
| --- | --- | --- |
| Parameter | r | p |
| Viral proportion respirable | 0.52096 | <.0001 |
| Host density | 0.27049 | <.0001 |
| Shedding magnitude | 0.26541 | <.0001 |
| Lower ID50 | -0.16025 | <.0001 |
| Surface area to volume ratio | -0.10241 | <.0001 |
| Viral inactivation in air | -0.08623 | <.0001 |
| Lung deposition fraction | 0.06828 | <.0001 |
| Breathing event rate | 0.03733 | <.0001 |
| Viral proportion inspirable | 0.00894 | 0.1905 |
| Self inoculation rate | -0.00777 | 0.255 |
| Medium particle settling rate | -0.00716 | 0.2945 |
| Hand-surface transfer efficiency | -0.00444 | 0.5155 |
| Upper ID50 | -0.0044 | 0.5198 |
| Viral inactivation on surfaces | -0.00384 | 0.5738 |
| Proportion of virus transported to target membrane | 0.00211 | 0.7578 |
| Viral inactivation on skin | 0.00206 | 0.7629 |
| Surface touching rate | 0.00189 | 0.7824 |
| Movement rate | -0.00069 | 0.9192 |

| Inspiration Infection |  |  |
| --- | --- | --- |
| Parameter | r | p |
| Upper ID50 | -0.52203 | <.0001 |
| Shedding magnitude | 0.42527 | <.0001 |
| Host density | 0.19252 | <.0001 |
| Viral proportion inspirable | 0.05641 | <.0001 |
| Hand-surface transfer efficiency | -0.01146 | 0.0858 |
| Viral inactivation on surfaces | -0.00799 | 0.2311 |
| Proportion of virus transported to target membrane | -0.00784 | 0.2399 |
| Self inoculation rate | -0.00777 | 0.2441 |
| Medium particle settling rate | -0.00591 | 0.3753 |
| Viral inactivation in air | 0.00564 | 0.3977 |
| Movement rate | -0.00308 | 0.6447 |
| Surface area to volume ratio | -0.00274 | 0.7329 |
| Viral proportion respirable | 0.00252 | 0.706 |
| Viral inactivation on skin | 0.0024 | 0.7185 |
| Lower ID50 | -0.00228 | 0.732 |
| Breathing event rate | 0.00218 | 0.7441 |
| Lung deposition fraction | -0.00195 | 0.7705 |
| Surface touching rate | -0.00096 | 0.8859 |

| Droplet Infection |  |  |
| --- | --- | --- |
| Parameter | r | p |
| Host density | 0.4439 | <.0001 |
| Upper ID50 | -0.39371 | <.0001 |
| Shedding magnitude | 0.33395 | <.0001 |
| Viral inactivation in air | 0.0109 | 0.1031 |
| Proportion of virus transported to target membrane | -0.01035 | 0.1217 |
| Viral inactivation on surfaces | -0.00826 | 0.2169 |
| Hand-surface transfer efficiency | -0.00815 | 0.2231 |
| Movement rate | -0.00508 | 0.4477 |
| Breathing event rate | 0.00458 | 0.4935 |
| Surface area to volume ratio | 0.00402 | 0.6175 |
| Self inoculation rate | -0.00381 | 0.5688 |
| Viral inactivation on skin | 0.0037 | 0.5804 |
| Viral proportion inspirable | 0.00256 | 0.7024 |
| Medium particle settling rate | 0.00153 | 0.8193 |
| Surface touching rate | 0.00095 | 0.8875 |
| Lower ID50 | -0.00008 | 0.9902 |
| Lung deposition fraction | -0.00005 | 0.9946 |
| Viral proportion respirable | 0.00004 | 0.995 |

| Contact Infection |  |  |
| --- | --- | --- |
| Parameter | r | p |
| Upper ID50 | -0.27871 | <.0001 |
| Viral inactivation on skin | -0.2662 | <.0001 |
| Shedding magnitude | 0.25751 | <.0001 |
| Hand-surface transfer efficiency | 0.23128 | <.0001 |
| Proportion of virus transported to target membrane | 0.18915 | <.0001 |
| Surface touching rate | 0.16394 | <.0001 |
| Self inoculation rate | 0.15122 | <.0001 |
| Host density | 0.13211 | <.0001 |
| Viral inactivation on surfaces | -0.09667 | <.0001 |
| Breathing event rate | -0.01369 | 0.041 |
| Medium particle settling rate | 0.01357 | 0.0428 |
| Movement rate | 0.01295 | 0.0532 |
| Viral inactivation in air | 0.01156 | 0.0845 |
| Viral proportion inspirable | 0.00895 | 0.1814 |
| Lower ID50 | 0.00498 | 0.4569 |
| Viral proportion respirable | -0.00413 | 0.5373 |
| Lung deposition fraction | -0.00348 | 0.6031 |
| Surface area to volume ratio | 0.00112 | 0.8896 |

1. Gillespie DT (1977) Exact stochastic simulation of coupled chemical reactions. The Journal of Physical Chemistry 81: 2340-2361. doi:10.1021/j100540a008

2. Anderson RM, May RM, Anderson B (1992) Infectious Diseases of Humans: Dynamics and Control. Oxford University Press, USA.

3. Diekmann O, Heesterbeek JAP, Metz JAJ (1990) On the definition and the computation of the basic reproduction ratio R0 in models for infectious diseases in heterogeneous populations. Journal of Mathematical Biology 28: 365-382. doi:10.1007/BF00178324

4. Hayden FG, Fritz R, Lobo MC, Alvord W, Strober W, et al. (1998) Local and systemic cytokine responses during experimental human influenza A virus infection. Relation to symptom formation and host defense. J. Clin. Invest 101: 643-649. doi:10.1172/JCI1355

5. Nicas M, Nazaroff WW, Hubbard A (2005) Toward understanding the risk of secondary airborne infection: emission of respirable pathogens. J Occup Environ Hyg 2: 143-154. doi:10.1080/15459620590918466

6. Loudon RG, Spohn SK (1969) Cough frequency and infectivity in patients with pulmonary tuberculosis. Am. Rev. Respir. Dis 99: 109-111.

7. Loudon RG, Roberts RM (1967) Droplet expulsion from the respiratory tract. Am. Rev. Respir. Dis 95: 435-442.

8. Duguid JP (1946) The Size and the Duration of Air-Carriage of Respiratory Droplets and Droplet-Nuclei. The Journal of Hygiene 44: 471-479.

9. HEMMES JH, WINKLER KC, KOOL SM (1960) Virus survival as a seasonal factor in influenza and polimyelitis. Nature 188: 430-431.

10. Bean B, Moore BM, Sterner B, Peterson LR, Gerding DN, et al. (1982) Survival of Influenza Viruses on Environmental Surfaces. The Journal of Infectious Diseases 146: 47-51.

11. Sattar S, S. Springthorpe, S. Mani, M. Gallant, R. C. Nair, et al. (2001) Transfer of bacteria from fabrics to hands and other fabrics: development and application of a quantitative method using <i>Staphylococcus aureus</i> as a model. Journal of Applied Microbiology 90: 962-970. doi:10.1046/j.1365-2672.2001.01347.x

12. Rheinbaben FV, Schünemann S, Groß T, Wolff MH (2000) Transmission of viruses via contact in ahousehold setting: experiments using bacteriophage [phi]X174 as a model virus. Journal of Hospital Infection 46: 61-66. doi:10.1053/jhin.2000.0794

13. Hendley JO, Wenzel RP, Gwaltney JM (1973) Transmission of rhinovirus colds by self-inoculation. N. Engl. J. Med 288: 1361-1364.

14. Nicas M, Best D (2008) A study quantifying the hand-to-face contact rate and its potential application to predicting respiratory tract infection. J Occup Environ Hyg 5: 347-352. doi:10.1080/15459620802003896

15. Rusin P, S. Maxwell, C. Gerba (2002) Comparative surface-to-hand and fingertip-to-mouth transfer efficiency of gram-positive bacteria, gram-negative bacteria, and phage. Journal of Applied Microbiology 93: 585-592. doi:10.1046/j.1365-2672.2002.01734.x

16. US EPA National Center for Environmental Assessment WD, Jacqueline Moya (2000) Exposure Factors Handbook (Final Report) 1997. Available: http://cfpub.epa.gov/ncea/cfm/recordisplay.cfm?deid=12464&CFID=6224637&CFTOKEN=49828852&jsessionid=5a30136899d3d95cee9a195c6646342f7b10. Accessed 2 Nov 2009.

17. ICRP, ICRP (1995) ICRP Publication 66: Human Respiratory Tract Model for Radiological Protection. 1st ed. Elsevier.

18. Alford RH, Kasel JA, Gerone PJ, Knight V (1966) Human influenza resulting from aerosol inhalation. Proc. Soc. Exp. Biol. Med 122: 800-804.

19. Atkinson M, Wein L (2008) Quantifying the Routes of Transmission for Pandemic Influenza. Bulletin of Mathematical Biology 70: 820-867. doi:10.1007/s11538-007-9281-2

20. Couch RB, Douglas RG, Fedson DS, Kasel JA (1971) Correlated studies of a recombinant influenza-virus vaccine. 3. Protection against experimental influenza in man. J. Infect. Dis 124: 473-480.

21. Couch RB, Kasel JA, Gerin JL, Schulman JL, Kilbourne ED (1974) Induction of partial immunity to influenza by a neuraminidase-specific vaccine. J. Infect. Dis 129: 411-420.

22. Wallace L (1996) Indoor particles: a review. J Air Waste Manag Assoc 46: 98-126.

23. Riley WJ, McKone TE, Lai ACK, Nazaroff WW (2002) Indoor particulate matter of outdoor origin: importance of size-dependent removal mechanisms. Environ. Sci. Technol 36: 200-207.

24. Hong T (2009) Estimating Risk of Exposure to Bacillus Anthracis based on Environmental Concentrations. Drexel University. Available: http://hdl.handle.net/1860/3012. Accessed 3 Nov 2009.
